# Targeting CIC::DUX4 sarcoma with Minnelide in a dual recombinase–initiated genetically engineered mouse model

**DOI:** 10.1172/JCI202218

**Published:** 2026-06-16

**Authors:** MaKenna R. Browne, Axel V. Silver, Risha Banerjee, Brendan C. Dickson, Benigno Aquino, Kristianne M. Oristian, Jonathon Himes, Peter G. Hendrickson, David G. Kirsch

**Affiliations:** 1 Cell and Molecular Biology Program, and; 2 Developmental and Stem Cell Biology Program, Duke University Medical Center, Durham, North Carolina, USA.; 3 Department of Radiation Oncology,; 4 Department of Medical Biophysics, and; 5 Department of Laboratory Medicine and Pathobiology, University of Toronto, Toronto, Ontario, Canada.; 6 Department of Pathology and Laboratory Medicine, Mount Sinai Hospital, Toronto, Ontario, Canada.; 7 Department of Radiation Oncology, Duke University Medical Center, Durham, North Carolina, USA.; 8 Radiation Medicine Program, Princess Margaret Cancer Centre, University Health Network, Toronto, Ontario, Canada.

**Keywords:** Cell biology, Oncology, Drug screens, Mouse models, Transcription

## Abstract

CIC::DUX4 sarcoma (CDS) is a lethal cancer driven by a fusion between the tumor suppressor capicua (CIC) and the pioneer transcription factor double homeobox 4 (DUX4). We previously generated 3 genetically engineered mouse models (GEMMs) of CDS with *CIC::DUX4* regulated by loxP-STOP-loxP cassettes, however, mice from all 3 models developed spontaneous tumors without Cre recombinase. Here, we established a next-generation GEMM of CDS (dual-flex [dFLEx] CDS) that used a dual recombinase (Cre plus the thermostable mutant of FLP recombinase FLPE) FLEx-switch design to activate *CIC::DUX4* expression and initiate sarcomagenesis in a spatially and temporally controlled manner. Because CIC::DUX4 drives sarcoma development by activating an oncogenic transcriptional program, we performed a drug screen on human-derived CDS cell lines using a library of compounds that modulate transcription. This screen identified Minnelide, an inhibitor of RNA polymerase II–mediated transcription, as a selective inhibitor of CDS. Mechanistically, Minnelide acted through xeroderma pigmentosum type B to alter phosphorylation of RPB1, the largest subunit of RNA polymerase II. Subsequently, RPB1 underwent degradation leading to apoptosis of CDS cells. Minnelide demonstrated in vivo efficacy in dFLEx CDS GEMMs and in human CDS xenografts. As Minnelide has already been demonstrated to be safe in clinical trials, these findings identify Minnelide as a potential therapeutic option to test in patients with CDS.

## Abstract 2

A next-generation mouse model helps identify Minnelide as a potential treatment for CIC::DUX4 sarcoma, a rare and deadly cancer with no treatment options

## Graphical abstract

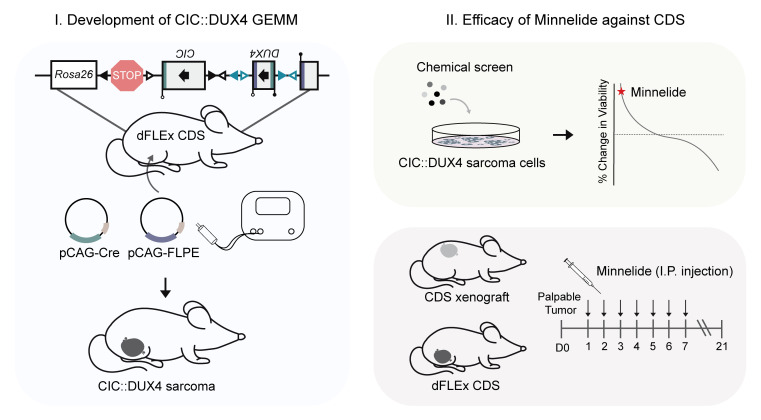

## Introduction

Capicua-rearranged (CIC-rearranged) sarcoma is a soft tissue sarcoma that primarily affects adolescents and young adults and is most commonly driven by t(4;19) or t(10;19) chromosomal translocations, resulting in a CIC:double homeobox 4 fusion (CIC::DUX4) (1–4). Until recently, CIC::DUX4 sarcoma (CDS) was referred to as Ewing-like sarcoma, given the similarity between the histological and immunohistochemical features of CDS and Ewing sarcoma. However, unlike Ewing sarcoma, CDS rarely arises in the bone, lacks the Ewing-defining ETS gene fusion, and is associated with a more aggressive clinical course. Although the WHO recently classified CIC-rearranged sarcomas as a distinct entity (5), patients are still routinely treated with Ewing sarcoma–based chemotherapy despite mounting data demonstrating limited efficacy, high rates of recurrence, and poor outcomes (2, 3, 6). Therefore, alternative treatments that are effective in patients with CDS are urgently needed.

Mechanistically, CDS is driven by a unique fusion protein composed of the majority of the transcriptional repressor capicua, including its DNA binding domains, and the C-terminal transactivation domain of the pioneer transcription factor DUX4 (7). Endogenous CIC represses the expression of genes downstream of receptor tyrosine kinase activation (8–11), however, upon fusion to the C-terminal transactivation domain of DUX4, CIC::DUX4 functions as a potent transcriptional activator at normally repressed CIC binding sites (7, 12–14). Given that CDS is fundamentally driven by activation of an aberrant transcriptional program, modulating transcription could serve as a promising therapeutic approach.

A critical barrier to identifying better therapeutic options for CDS is the lack of robust preclinical models for identifying and testing alternative therapies. While many studies have successfully utilized patient-derived and ectopically derived xenograft models (12, 13, 15–20) to interrogate key oncogenic mechanisms of CDS, these models are limited by the absence of an intact immune system and the natural tumor microenvironment that is critical for tumor growth and response to therapy. Previous work reported on the development of a transgenic CIC::DUX4 sarcoma zebrafish model (21), however, generation of an immunocompetent, genetically engineered mouse model (GEMM) has been challenging. Our first attempts to generate GEMMs involved the use of loxP-STOP-loxP cassettes and relied on the expression of Cre recombinase to activate the expression of *CIC::DUX4* either at the *Rosa26* locus or at the endogenous *Cic* locus (22). Notably, in the absence of Cre, mice from all 3 models developed CDS tumors spontaneously and progressed rapidly before the mice were able to breed, preventing colony maintenance (22). Importantly, a recent study demonstrated that doxycycline-inducible *CIC::DUX4* chimeric mice can be utilized to gain temporal control over tumor formation (23), however, alternative approaches are necessary to generate both a spatially and temporally controllable CIC::DUX4 sarcoma mouse model.

In this study, we developed a next-generation GEMM of CDS (dual-flex [dFLEx] CDS) that involved the use of 2 independent recombinases (Cre plus the thermostable mutant of FLP recombinase FLPE) to activate *CIC::DUX4* expression in a controlled manner. Furthermore, we conducted a drug screen on human-derived CDS cell lines using a library of compounds that modulate transcription including drugs that selectively target a diverse array of epigenetic writers and erasers. By leveraging our in vitro drug study, human CDS xenografts, and dFLEx CDS GEMMs, we demonstrate that Minnelide, an inhibitor of RNA pol II transcription, is a promising therapeutic approach for CDS.

## Results

### Dual-recombinase regulation of CIC::DUX4 expression results in a spatially and temporally restricted CDS mouse model.

**Figure 1:**
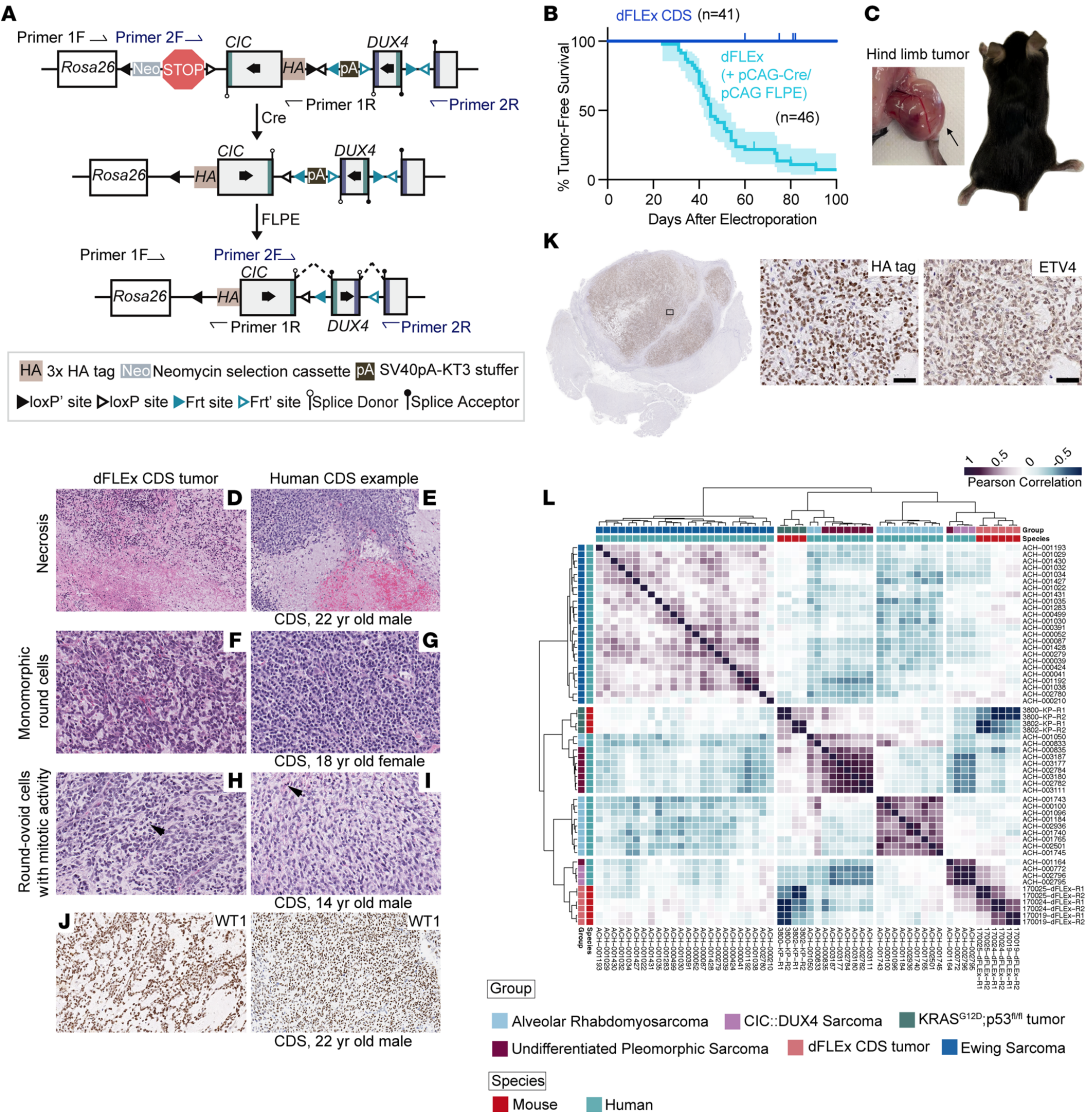
dFLEx CDS mice develop tumors only after expression of Cre and FLPE. (**A**) Schematic of the dFLEx CDS allele before and after recombination with Cre and FLPE. PCR primer sequences used to assess recombination (Supplemental Figure 1L) are indicated with Primer 1F/R and Primer 2F/R arrows. (**B**) Kaplan-Meier survival curve of dFLEx CDS mice showing no spontaneous tumor development without Cre and FLPE and approximately 90% tumor penetrance after electroporation of the hind limb muscle with plasmids expressing Cre plus FLPE. (**C**) Gross images of a hind limb tumor. (**D**–**J**) Representative photomicrographs highlighting histologic and immunohistochemical similarity between dFLEx CDS model and clinical examples of CIC::DUX4 sarcoma. (**D** and **E**) Necrosis in tumors originating from a dFLEx CDS mouse and the foot of a 22-year-old male individual (H&E; original magnification, ×100). (**F** and **G**) Sheets of monomorphic round cells, with occasional interspersed epithelioid cells, originating from a dFLEx CDS mouse and the shoulder of an 18-year-old female individual (H&E; original magnification, ×400). (**H** and **I**) Vague fascicles of monomorphic, round-ovoid cells with mitotic activity (arrowheads) and myxoid stroma originating from dFLEx CDS mouse and the thigh of a 14-year-old male individual (H&E; original magnification, ×400). (**J**) Diffuse nuclear staining for WT1 in cells from a dFLEx CDS mouse and a 22-year-old male individual (IHC; original magnification, ×200). (**K**) IHC of tumors formed in dFLEx CDS mice after electroporation of Cre plus FLPE plasmids for HA tag and ETV4 (scale bars: 50 μm). Supplemental Figure 1A shows an expanded staining panel; representative ETV4 and HA tag images from **K** are included again for completeness. (**L**) Pearson correlation of dFLEx CDS and *KRAS^G12D^ Trp53^fl/fl^* (KP) tumor transcriptional profiles to fusion-positive human sarcoma cell lines.

A barrier to preclinical investigation of alternative therapeutic approaches for CDS is the lack of an immunocompetent, autochthonous mouse model. Our laboratory previously attempted to develop a GEMM of CDS by targeting mouse embryonic stem (ES) cells using 3 different approaches that each regulated *CIC::DUX4* expression with loxP-STOP-loxP cassettes (22). Remarkably, mice derived from the ES cells in all 3 models spontaneously developed aggressive and multifocal sarcomas in the absence of Cre recombinase, leading to rapid and early animal demise (22). Therefore, in these models, we were unable to control the timing or location of tumor formation, and it was not possible to breed the transgenic allele. To address these limitations, we developed a fourth CDS mouse model in which *CIC::DUX4* expression required both Cre and FLP recombinases to conditionally invert 2 exons into the correct orientation and reading frame (Figure 1A). In the absence of Cre and FLP recombinases, a neomycin STOP cassette precedes an inverted exon 1 (*CIC*), SV40pA-KT3 stuffer sequence (pA), and inverted exon 2 (*DUX4*). Cre-mediated recombination inverts exon 1 into the same orientation as the CAG promoter and excises the neomycin STOP cassette. Following Cre recombination, the SV40pA-KT3 stuffer sequence remains intact, and the inverted exon 2 is out of frame. FLP-mediated recombination excises the SV40pA-KT3 stuffer sequence and inverts exon 2 into the same orientation and in frame with the remainder of the *CIC::DUX4* transcript. Both Cre and FLP-mediated recombination are therefore required for *CIC::DUX4* transgene expression (Figure 1A). Indeed, in the absence of Cre and FLP, the dFLEx CDS system prevented spontaneous tumor formation. Following electroporation of pCAG-Cre and pCAG-FLPE plasmids into the hind limbs of heterozygous and homozygous dFLEx CDS mice, spatially restricted tumors formed within 3 months at approximately 90% penetrance (Figure 1, B and C). H&E-stained sections (Figure 1, D–I, and Supplemental Figure 1, A–I; supplemental material available online with this article; https://doi.org/10.1172/JCI202218DS1) showed morphologically small, round cell tumors with strong expression of WT1, HA tag, and ETV4 (Figure 1, J and K) and patchy and/or focal immunoreactivity for desmin and CD99 (Supplemental Figure 1A); pancytokeratin was negative (Supplemental Figure 1A). Overall, the findings were broadly analogous to human CIC::DUX4 fusion–positive tumors (Figure 1, D–J). Furthermore, RNA-seq comparing the transcriptional profiles of the dFLEx CDS tumors versus sarcomas in *KRAS^loxP-STOP-loxP-G12D^* Tr*p53^fl/fl^* (KP) mice demonstrated that dFLEx CDS tumors expressed known CIC::DUX4 target genes (14, 22) such as *Etv1/4/5*, *Dusp6*, *Shc3/4*, and *Spred3* (Supplemental Figure 1J). To assess whether dFLEx CDS tumors transcriptionally recapitulate human CDS, we conducted a cross-species transcriptomics comparison to evaluate the similarity between dFLEx CDS tumors and human sarcoma cell lines from the Cancer Dependency Map (DepMap) database. Remarkably, we found that dFLEx CDS tumors clustered together with the human CDS cell lines (Figure 1L), suggesting that dFLEx CDS tumors were transcriptionally similar to human CDS. Additionally, GSEA analysis revealed a strong correlation between dFLEx CDS tumors and human CDS cell lines (*r* = 0.87) in the enrichment of several notable driver oncogenic signaling pathways including MYC targets, mTOR, Notch, PI3K/AKT, and TGF-β signaling (Supplemental Figure 1K). PCR amplification across the loxP and FRT sites confirmed successful recombination by Cre and FLPE, respectively (Supplemental Figure 1L), and the CIC::DUX4 fusion protein was detected in dFLEx CDS tumors by Western blotting with antibodies against HA tag and DUX4 (Supplemental Figure 1M). Collectively, these results demonstrate that the use of 2 independent recombinase systems prevented spontaneous tumor formation in the absence of a recombinase, whereas electroporation of separate pCAG-Cre and pCAG-FLPE plasmids enabled spatially and temporally restricted sarcomas that mimicked human CDS.

### Single-nucleus RNA-seq of dFLEx CDS tumors.

**Figure 2:**
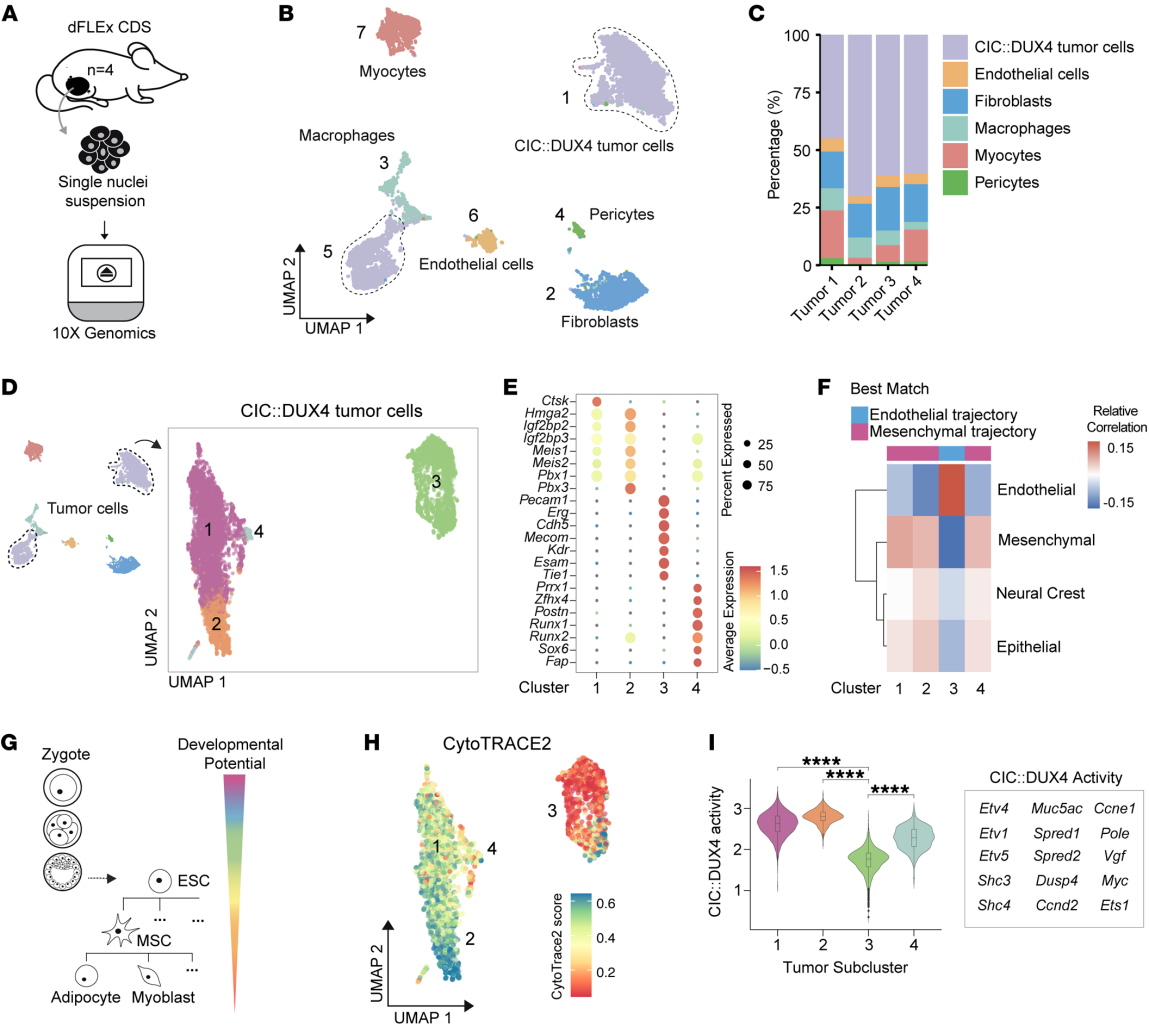
Single-nucleus transcriptional profiling of dFLEx CDS tumors. (**A**) Schematic of snRNA-seq workflow. Single nuclei suspensions for snRNA-seq were prepared from 4 dFLEx CDS tumors. (**B**) UMAP plots of tumor and nontumor cells (*n* = 15,923) in dFLEx CDS tumors (*n* = 4). Cell types were assigned using the expression of canonical marker genes (Supplemental Figure 2B). (**C**) Bar plots showing the cluster composition in each tumor. (**D**) UMAP plot of tumor cells only. (**E**) Dot plot highlighting marker gene expression in tumor subclusters. (**F**) Heatmap demonstrating the correlation between tumor subclusters and MOCA developmental trajectories. (**G**) Adapted schematic of the CytoTRACE2 deep learning model (29). CytoTRACE2 predicts cellular potency and developmental potential based on gene expression profiles. (**H**) UMAP of CytoTRACE2 scores. Clusters with higher CytoTRACE2 scores are predicted to have higher differentiation potential and be less differentiated. (**I**) CIC::DUX4 activity in each tumor subcluster based on the expression of CIC::DUX4 target genes and genes highly expressed in CDS tumors. Brackets indicate significance by 1-sided Wilcoxon rank-sum test with Benjamini-Hochberg (BH) correction. Effect sizes (rank-biserial *r*): cluster 3 vs. 1, *r* = 0.97; vs. 2, *r* = 1.00; vs. 4, *r* = 0.85. ****P < 0.0001.

Next, to better characterize intertumoral properties and the tumor microenvironment, we conducted single nucleus RNA-seq (snRNA-seq) on 4 dFLEx CDS tumors using 10X Genomics Sequencing (Figure 2A). After quality control and doublet removal, a total of 15,923 cells were retained for unsupervised clustering on the basis of the top variably expressed genes (Figure 2B and Supplemental Figure 2A). Given the expression of CIC::DUX4 target genes *Etv1*, *Etv4*, *Etv5*, *Shc3*, *Shc4*, *Dusp4*, *Vgf*, and *Spred2* (Supplemental Figure 2, B and C), clusters 1 and 5 were annotated as CIC::DUX4 tumor cells, whereas the remaining clusters were assigned using canonical marker genes (Supplemental Figure 2B) and corresponded to fibroblasts, pericytes, macrophages, endothelial cells, and myocytes (Figure 2B). The relative proportions of each cluster were broadly similar across all 4 tumors, with CIC::DUX4 sarcoma cells comprising the majority (45%–70%) of cells within each tumor (Figure 2C).

To further investigate characteristics of the CIC::DUX4 tumor cells, tumor cell clusters 1 and 5 were subclustered, revealing 4 subclusters (Figure 2D and Supplemental Figure 2D). Subcluster-level differential expression analysis (FDR < 0.05, log fold change [FC] > 0.5) revealed that cluster 2 was enriched for genes associated with self-renewal and stemness, cluster 3 was strongly enriched for endothelial cell–associated genes such as *Pecam1*, *Erg*, and *Cdh5*, and cluster 4 was enriched for mesenchymal cell–associated genes (Figure 2E). Comparison of the tumor cell transcriptional profiles to the Mouse Organogenesis Cell Atlas (MOCA) reference dataset (24) revealed subclusters 1, 2, and 4 most closely correlated to the mesenchymal trajectory, whereas cluster 3 strongly correlated to the endothelial cell trajectory (Figure 2F). Interestingly, a single-cell study of human CDS tumors also recently reported tumor cell populations with endothelial cell–like transcriptional features (25), and clinically, there are several reports of histologically unique CIC::DUX4 sarcomas with angiosarcoma-like features including ERG/CD31 expression (26–29).

Given the histologic undifferentiated nature of both human CDS and dFLEx CDS tumors, we next wondered if we could make inferences about the cellular potency and developmental potential of the different tumor subclusters. Using the deep learning model CytoTRACE2 (30) to characterize the developmental state of dFLEx CDS tumor cells (Figure 2G), we found that most tumor cells had moderate CytoTRACE2 scores. However, cells in subcluster 2 had higher scores, whereas cells in subcluster 3 had lower scores (Figure 2H), suggesting diversity in developmental potential across tumor cell populations. Notably, tumor cluster 5, which gives rise to tumor subcluster 3, displayed lower activation of the CIC::DUX4 transcriptional program when compared with cluster 1 (Supplemental Figure 2, B and C). Given these observations, we next wondered if there is an association between developmental potential and CIC::DUX4 activity. To assess this, we generated a CIC::DUX4 activity score based on known CIC::DUX4 target genes and found that, indeed, the tumor subcluster with the lowest CytoTRACE2 score (subcluster 3) also had reduced CIC::DUX4 activity (Figure 2I).

### Minnelide kills CIC::DUX4 sarcoma cells through induction of apoptosis.

**Figure 3:**
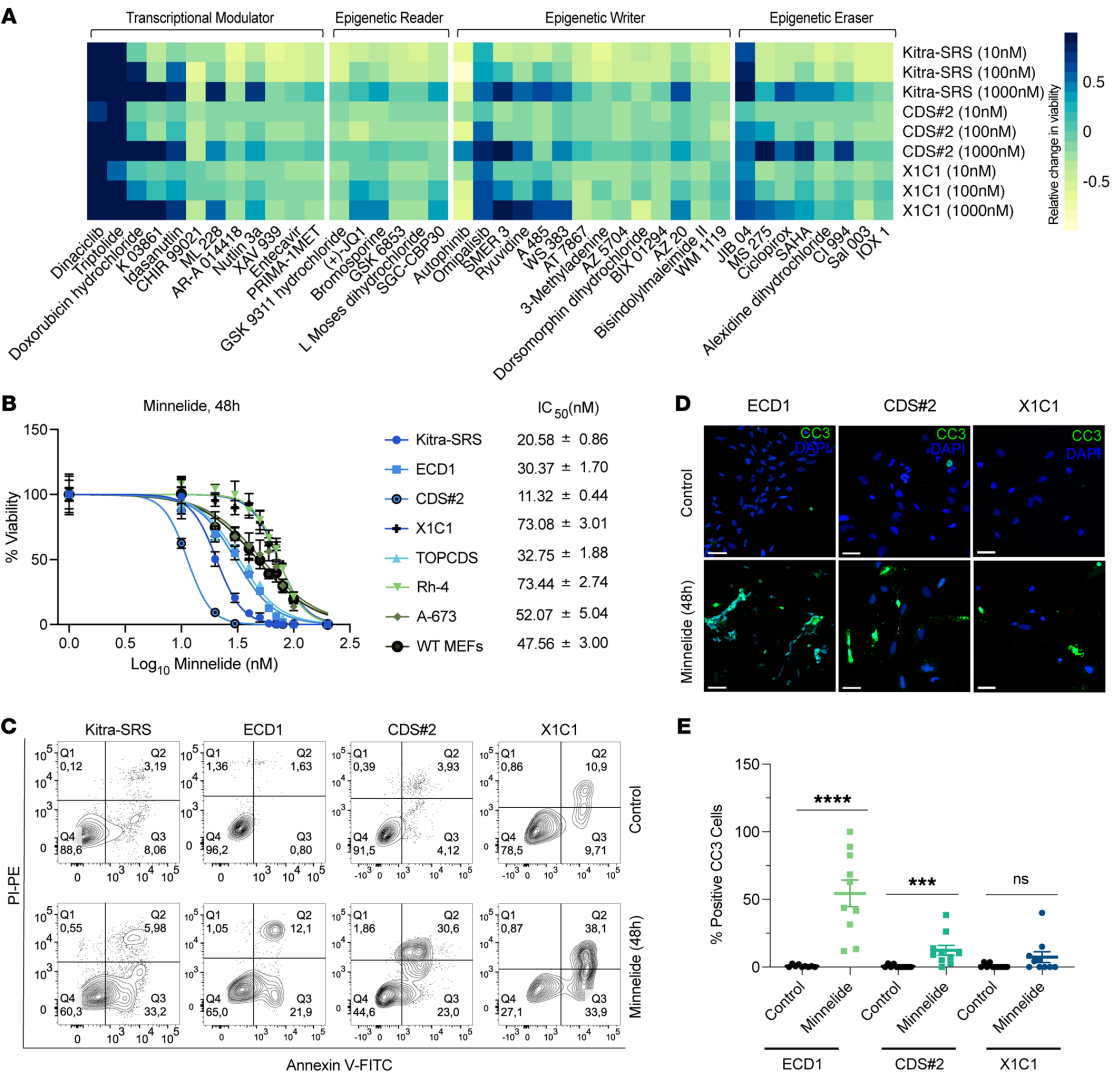
Minnelide induces apoptosis in CDS cells. (**A**) Viability drug screen on human CDS cells (Kitra-SRS, CDS#2 and X1C1) using the Tocriscreen Epigenetics 3.0 compound library. (**B**) CellTiter-Glo on CDS cells (Kitra-SRS, ECD1, CDS#2, X1C1, and TOPCDS mouse CDS cell lines [ref. 22]) and non-CDS cells (Rh-4, A-673, WT MEFs) treated with 0–200 nM Minnelide for 48 hours. (**C**) Annexin V/PI flow cytometry on Kitra-SRS, ECD1, CDS#2, and X1C1 CDS cells treated with Minnelide for 48 hours. (**D**) CC3 immunofluorescence of human CDS cells (ECD1, CDS#2, X1C1) treated with Minnelide for 48 hours. Scale bars: 50 μm. (**E**) Quantification of the percentage of CC3^+^ cells. A total of 9–10 regions per condition were quantified, including regions sampled from 3 independent experiments. ***P < 0.001 and ****P < 0.0001, by 2-tailed exact Mann-Whitney *U* test. Data are presented as the mean ± SEM.

Following the development of the dFLEx CDS model, we aimed to identify and evaluate alternative therapeutic interventions for CDS using this platform. Considering that CIC::DUX4 interacts with acetyltransferase p300/CBP to activate a unique oncogenic transcriptional program (14, 16), we sought to test the efficacy of compounds that modulate transcription or selectively target epigenetic writers and erasers. To this end, we performed cell viability screens in 3 human CDS cell lines (Kitra-SRS, CDS2, X1C1) at 4 concentrations (1 nm, 10 nm, 100 nm, and 1,000 nM) using a library of 160 small-molecule inhibitors (Figure 3A and Supplemental Figure 3A). This drug screen not only identified dinaciclib, a transcriptional modulator previously shown to have efficacy in CDS models (12), but also revealed a sensitivity to triptolide, starting in the 10 nM range (Figure 3A and Supplemental Figure 3A). Although triptolide has demonstrated robust antitumor effects in a variety of preclinical cancer models (31–41), the clinical utility of triptolide is limited by its poor solubility in water. Therefore, a water-soluble prodrug of triptolide called Minnelide was developed (42). Minnelide rapidly releases triptolide when exposed to phosphatases present in both tissues and in the blood in vivo (42) or in FBS in vitro. Importantly, Minnelide has been successfully tested in phase I and II clinical trials for advanced gastrointestinal (GI) carcinoma and pancreatic cancer (43, 44) and is currently being studied in ongoing clinical trials of gastric cancer (NCT05566834) and small-cell lung cancer (NCT05166616). To validate our screen results and to test the efficacy of Minnelide in CDS cells relative to other small, round-cell sarcoma cell lines, we performed CellTiter-Glo assays. After 48 hours of treatment, the CDS cell lines Kitra-SRS, ECD1, TOPCDS, and CDS#2 exhibited reduced viability compared with Ewing sarcoma (A-673), fusion-positive rhabdomyosarcoma (Rh-4), and WT mouse embryonic fibroblasts (WT MEFs) (Figure 3B). Notably, our drug screen revealed that triptolide reduced X1C1 CDS cell viability less effectively at 10 nM than in the other CDS cell lines included in the screen (Figure 3A). Similarly, the X1C1 cell line demonstrated lower sensitivity to Minnelide relative to the other CDS cell lines tested (Figure 3B). These results suggest that Minnelide and triptolide have similar efficacy patterns in human CDS cell lines.

Previous studies have shown Minnelide and triptolide induce apoptosis in some cancer cell types and autophagy in others (34–36, 39, 40, 45–48). To explore the mechanism of cell death in CDS cells, we treated Kitra-SRS, ECD1, and CDS#2 human CDS cells with 25 nM Minnelide for 48 hours and treated X1C1 human CDS cells with 70 nM Minnelide for 48 hours, and then performed annexin V/propidium iodide (PI) flow cytometry. In all cell lines tested, Minnelide treatment resulted in an increase of annexin V^+^/PI^–^ cells, indicating early apoptosis (Figure 3C). Additionally, after 48 hours, we observed an increase in cleaved caspase-3 (CC3) expression (Figure 3, D and E) and a decrease in EdU incorporation (Supplemental Figure 3B) further suggesting Minnelide induces apoptosis in CDS cells.

### Minnelide targets xeroderma pigmentosum type B, leading to RPB1 degradation and inhibition of transcription.

**Figure 4:**
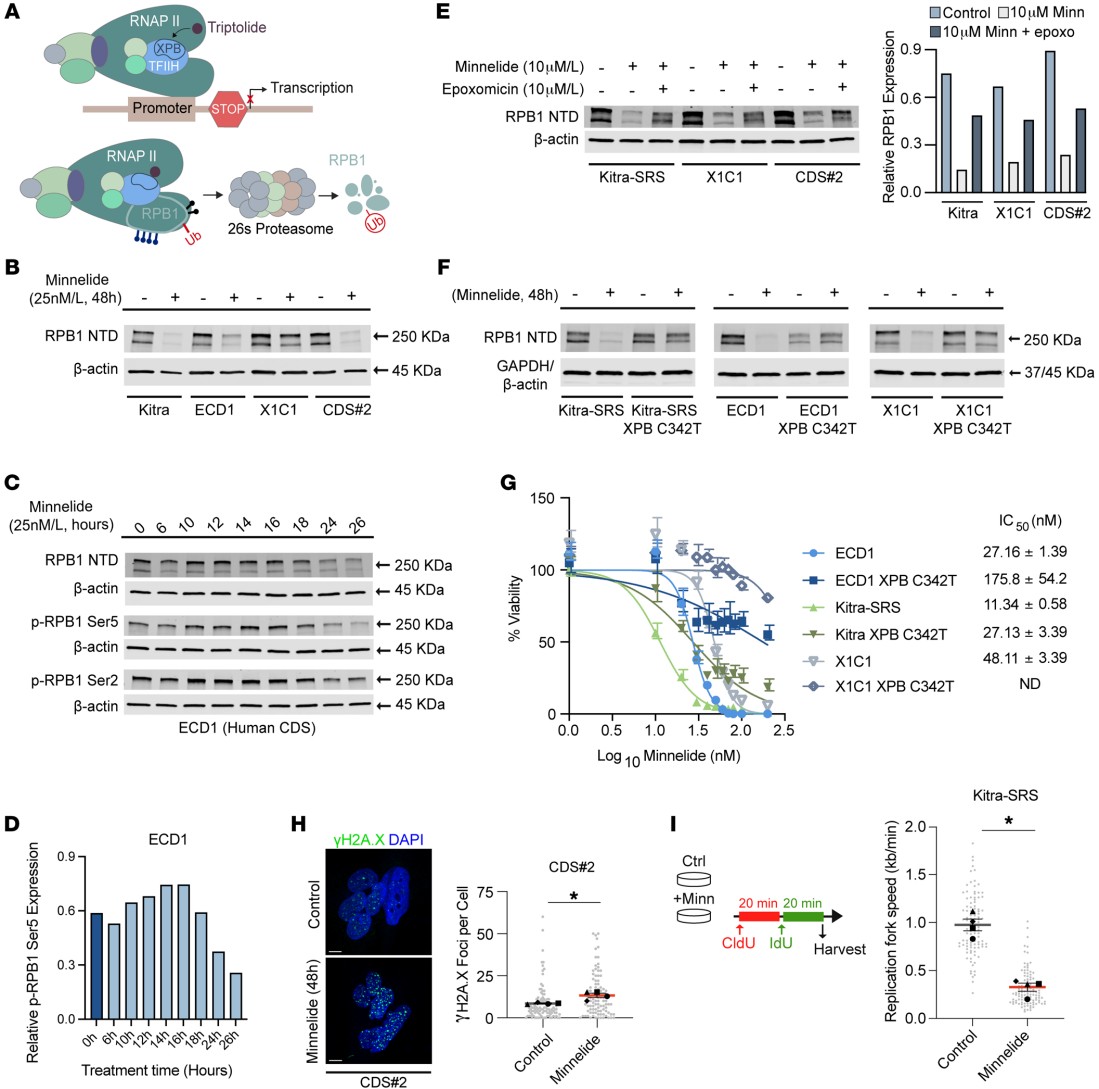
Minnelide targets XPB, leading to RPB1 degradation and transcriptional inhibition. (**A**) Schematic showing Minnelide inhibition of RNAP II. Minnelide directly binds XPB. Inhibition of XPB’s ATPase activity leads to stalling of RNAP II at gene promoters and inhibition of transcription. Prolonged stalling of RNAP II results in altered phosphorylation patterns on RPB1, ubiquitination, and then proteasome-mediated degradation of RPB1. (**B**) Western blot of RPB1 expression in human CDS cells (Kitra-SRS, ECD1, X1C1, and CDS#2) after treatment with 25 nM Minnelide for 48 hours. (**C**) Western blot of RPB1 expression and p-RPB1 over a time course of Minnelide treatment in human ECD1 cells. (**D**) Quantification of p-RPB1 (Ser5) expression (**E**) Western blot demonstrating that the proteasome inhibitor epoxomicin partially rescued Minnelide-mediated RPB1 degradation in CDS cells (Kitra-SRS, X1C1, CDS#2). (**F**) Western blot demonstrating that the expression of XPB C342T in Kitra-SRS, ECD1, and X1C1 human CDS cells rescued Minnelide-mediated RPB1 degradation. GAPDH was used as the loading control for Kitra-SRS and ECD1 cells, whereas β-actin was used as the loading control for X1C1 cells. (**G**) CellTiter-Glo assay demonstrated that human CDS cells expressing XPB C342T had increased resistance to Minnelide. (**H**) γH2A.X immunofluorescence on human CDS#2 cells treated with Minnelide for 48 hours. Scale bars: 5 μm. At least 50 nuclei per condition were quantified, including nuclei sampled from 4 independent experiments. *P < 0.05, by 2-tailed exact Mann-Whitney *U* test. (**I**) DNA fiber assay on human Kitra-SRS cells treated with Minnelide (Minn) for 48 hours. Ctrl, control. At least 100 fibers per condition were quantified, including fibers sampled from 4 independent experiments. *P < 0.05, by 2-tailed, exact Mann-Whitney *U* test. Data are presented as the mean ± SEM.

Previous studies have shown that Minnelide/triptolide directly binds to xeroderma pigmentosum type B (XPB), a subunit of transcription factor II H (TFIIH), which is a general transcription factor of RNA polymerase II (RNAP II) (37, 49–51). In this working model, upon binding, Minnelide inhibits XPB’s ATPase activity, leading to stalling of RNAP II at gene promoters and inhibition of sustained transcription. Prolonged stalling of RNAP II can result in altered phosphorylation patterns on RPB1, the largest subunit of RNAP II, leading to its ubiquitination and proteasome-mediated degradation (49–51) (Figure 4A). Consistent with this model, after treating human CDS cells with 25 nM Minnelide for 48 hours, we observed depletion of RPB1 in Kitra-SRS, ECD1, and CDS#2 human CDS cell lines (Figure 4B) and a near complete loss in the X1C1 cells after treating them with 70 nM Minnelide for 48 hours (Supplemental Figure 4A). Furthermore, after 6–26 hours of Minnelide treatment, we observed an initial increase in RPB1 phosphorylation at Ser5 (marking transcriptional initiation), whereas RPB1 phosphorylation at Ser2 (marking transcriptional elongation) was maintained (Figure 4, C and D, and Supplemental Figure 4B). By 26 hours, total RPB1 levels were reduced (Figure 4C and Supplemental Figure 4B). To determine whether the RPB1 reduction was due to proteasome-mediated degradation, CDS cells were cotreated with 10 mM Minnelide and 10 mM expoxomicin, a proteasome inhibitor. Cotreatment with epoxomicin partially rescued the Minnelide-mediated decrease in RPB1 expression (Figure 4E and Supplemental Figure 4C), suggesting that Minnelide treatment led to the proteasome-mediated degradation of RPB1 in CDS cells. Next, to test the model that Minnelide regulates RPB1 degradation via XPB, a Minnelide-resistant, XPB mutant (51) (XPB C342T) was expressed in human CDS cells (Supplemental Figure 4D). Human CDS cells expressing XPB C342T not only retained RPB1 expression after 48 hours of Minnelide treatment (Figure 4F) but also showed a higher tolerance to Minnelide across increasing concentrations (Figure 4G). Taken together, these results suggest that Minnelide targeted XPB, leading to RNAP II stalling and subsequent RPB1 degradation, thus inhibiting CDS cell viability.

A consequence of RNAP II stalling can be the accumulation of double-stranded breaks and an increase in transcription-replication conflicts leading to replication stress (52–55). Considering that CIC::DUX4 sarcoma cells use specific mechanisms to promote DNA repair and tolerance to elevated replication stress (12, 19, 56), we wondered if Minnelide treatment further increases DNA damage and replication stress, potentially contributing to Minnelide-mediated cell death. Forty-eight hours after Minnelide treatment, we observed an increase in γH2AX foci and protein expression (Figure 4H and Supplemental Figure 4, E and F), indicating an increase in DNA double-stranded breaks. Furthermore, DNA fiber analysis revealed that 48 hours of Minnelide treatment significantly decreased replication fork speed, indicating replication stress (Figure 4I and Supplemental Figure 4G).

### RPB1 loss coincides with the onset of Minnelide-induced cell death.

**Figure 5:**
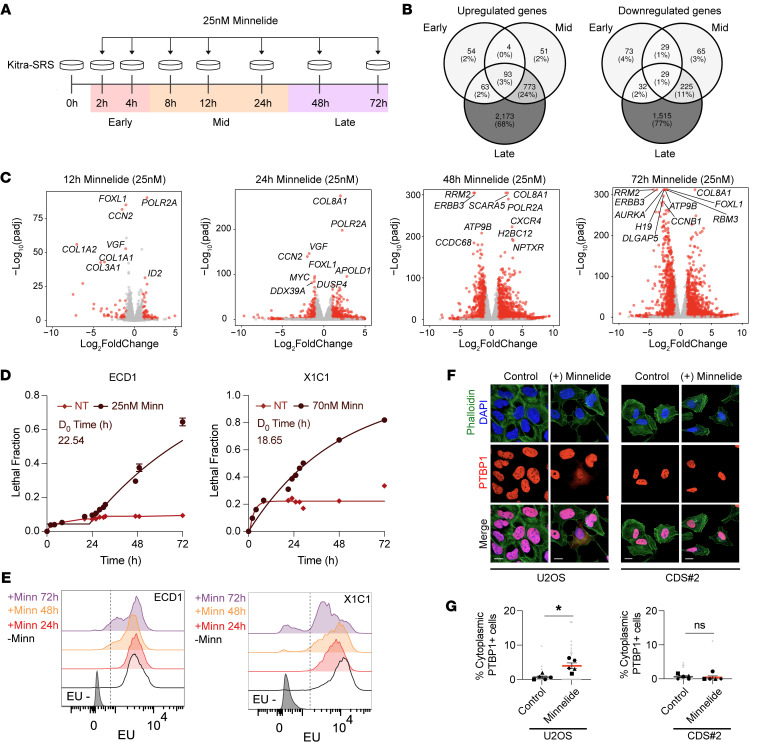
Minnelide induces cell death independent of transcriptional inhibition. (**A**) Schematic for the Minnelide time course in Kitra-SRS human CDS cells. (**B**) Venn diagrams representing transcriptional changes during early, mid, and late Minnelide treatment times. (**C**) Volcano plots demonstrating transcriptional changes following Minnelide treatment as a function of time. (**D**) FLICK assays on human ECD1 and X1C1 cells demonstrating that the onset of cell death was 22.54 hours and 18.65 hours after the start of Minnelide treatment, respectively. (**E**) EU incorporation 24, 48, and 72 hours after Minnelide treatment to assess changes in nascent transcription in ECD1 and X1C1 cells. (**F**) PTBP1 immunofluorescence on human CDS#2 cells after 48 hours Minnelide treatment. U2OS osteosarcoma cells were used as a positive control. Scale bars: 15 μm. (**G**) Quantification of cells with cytoplasmic PTBP1. A total of 24–25 regions per condition were quantified, including regions sampled from 5 independent experiments. *P < 0.05, by 2-tailed exact Mann-Whitney *U* test. Data are presented as the mean ± SEM.

Following RPB1 degradation, there are several potential pathways to induce cell death. One possibility is that inhibition of transcription leads to widespread mRNA decay over time, with progressive loss of essential proteins, which results in a passive form of cell death termed accidental cell death (57). A second possibility is that inhibition of transcription leads to the loss of expression of essential genes that are specifically regulated by CIC::DUX4, similar to G3 medulloblastoma (G3 MB), in which Minnelide-induced cell death occurs, in part, through an early (2–8 hours) reduction of the G3 MB dependency gene, *MYC* (37). Alternatively, a recent elegant study demonstrated that degradation of RPB1 itself can trigger apoptosis through a transcription-independent mechanism, termed the Pol II degradation–dependent apoptotic response (58). Given that CDS is dependent on an aberrantly activated transcriptional program, we initially hypothesized that, similar to what is seen with G3 MB, loss of expression of essential genes would be a key driver of cell death following Minnelide treatment. We started by treating human CDS cells (Kitra-SRS) with 25 nM Minnelide for 2, 4, 8, 12, 24, 48, and 72 hours and then conducted bulk RNA-seq (Figure 5A) to assess Minnelide-induced transcriptional changes over time. Notably, few transcriptional changes seemed to occur at the early treatment time points (2–4 hours); instead, most changes occurred between the mid-to-late treatment times (Figure 5B). Surprisingly, only a very small subset of CIC::DUX4 target genes emerged as top downregulated genes following 2–24 hours of Minnelide treatment. Target genes such as *DUSP4*, *VGF*, and *MYC* were downregulated at 24 hours, (Figure 5C), however, the majority of CDS target genes, including *ETV1/4/5*, remained relatively unchanged until 72 hours (Supplemental Figure 5A). In line with these results, CIC::DUX4 fusion expression was retained after both 24 and 48 hours of Minnelide treatment (Supplemental Figure 5B). Next, to evaluate if the late timing of target gene expression loss coincides with the onset of cell death, we conducted fluorescence-based and lysis-dependent inference of cell death kinetics (FLICK) (59) and determined that the onset of cell death occurred between 18 and 25 hours following the addition of Minnelide (Figure 5D and Supplemental Figure 5C). Furthermore, 5-ethynyl uridine (EU) incorporation 24, 48, and 72 hours after Minnelide treatment revealed that there were no major shifts in nascent transcription at 24 hours (Figure 5E and Supplemental Figure 5D), suggesting that the onset of cell death in CDS cells was not initially due to global loss of transcription. Instead, the onset of cell death closely coincided with the loss of RPB1 (Figure 4C and Supplemental Figure 4B), suggesting that RPB1 loss may have been triggering cell death in a transcription-independent manner (58). Previous studies in U2OS cells have found that degradation of RNAP II shifts the localization of BCL2L12 from the nucleus to mitochondria via a polypyrimidine tract–binding protein 1–dependent (PTBP1-dependent) mechanism to trigger the intrinsic pathway of apoptosis (58). While we were able to detect cytoplasmic translocation of PTBP1 in U2OS cells similar to a previous report (58), we did not detect PTBP1 translocation in human CDS cells after Minnelide treatment (Figure 5, F and G, and Supplemental Figure 5E). Collectively these results suggest that there are PTBP1-independent mechanisms triggering cell death following RPB1 degradation.

### Minnelide treatment significantly reduces tumor growth in human CDS xenograft models.

**Figure 6:**
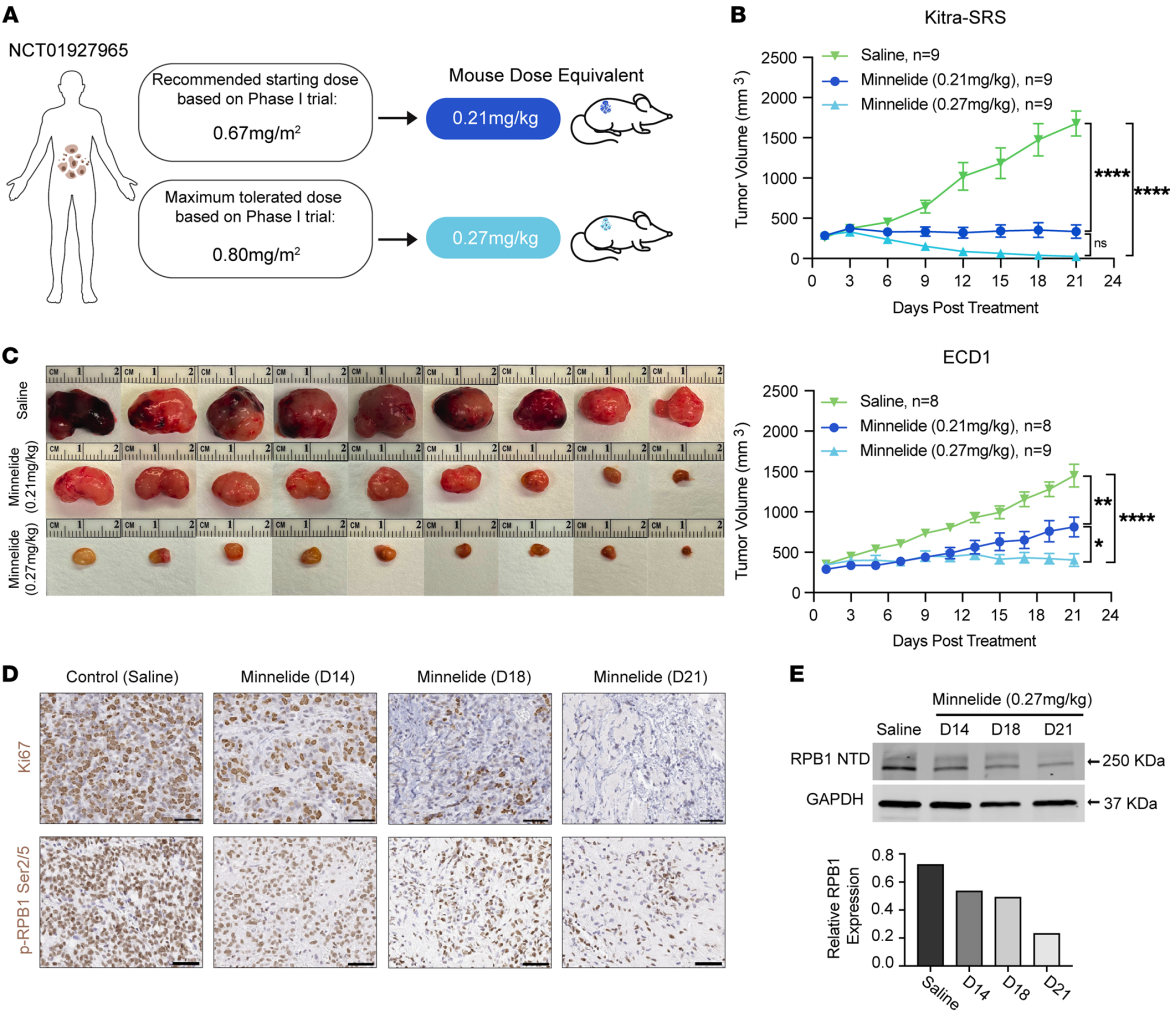
Minnelide reduces tumor growth in human CDS xenograft models. (**A**) Dose selection was informed by the recommended starting dose and maximum tolerated dose reported in the phase I Minnelide clinical trial for GI carcinoma (NCT01927965). Doses of 0.21 mg/kg and 0.27 mg/kg approximate the human doses of 0.67 mg/m^2^ and 0.80 mg/m^2^, respectively. (**B**) NSG mice were inoculated with Kitra-SRS or ECD1 human CDS cells and then treated with Minnelide at a dose of 0.21 mg/kg or 0.27 mg/kg daily for 21 days. Tumor growth was significantly reduced in the Minnelide-treated groups compared with controls. Data are presented as the mean ± SEM. *P < 0.05, **P < 0.01, and ****P < 0.0001, by 1-way ANOVA at day 21. (**C**) Gross morphology of Kitra-SRS dissected tumors at the humane endpoint or at day 21 of treatment. (**D**) Ki67 and p-RPB1 Ser2/5 IHC on sections from saline- and Minnelide-treated Kitra-SRS CDS xenograft tumors at 14, 18, and 21 days of Minnelide treatment. Scale bars: 50 μm. (**E**) Western blot of RPB1 expression in Kitra-SRS CDS xenograft tumors over a 21-day treatment period with 0.27 mg/kg Minnelide daily and quantification of RPB1 expression relative to GAPDH loading control. D14, day 14; D18, day 18; D21, day 21.

To test the clinical potential of Minnelide in CDS, human CDS cells (Kitra-SRS and ECD1) were s.c. transplanted into NOD-SCID IL2Rγ-null (NSG) mice. Once tumors were palpable (~200–300 mm^3^), mice were treated with Minnelide via i.p. injection once daily for 21 consecutive days. Dose selection was informed by the recommended starting dose and maximum tolerated dose reported in the phase I Minnelide clinical trial for GI carcinoma (43). Treatment with both 0.21 mg/kg and 0.27 mg/kg Minnelide, which approximates (60) the human dose of 0.67 mg/m^2^ and 0.80 mg/m^2^ (Figure 6A), significantly reduced tumor growth of xenografts from both of the human CDS cell lines tested (Figure 6, B and C, and Supplemental Figure 6A). Notably, a 21-day treatment course with 0.27 mg/kg resulted in sustained tumor control, with no recurrence observed through 50 days (Supplemental Figure 6, B and C).

Immunohistochemical staining on Minnelide xenograft tumor tissues demonstrated a reduction in Ki67 staining over the 21-day treatment period, and reduced expression of phosphorylated RPB1 (p-RPB1) at Ser2/5, indicating decreased RNAP II initiation and elongation and suggesting that active transcription was impaired (Figure 6D). Furthermore, Western blot analysis of xenograft tumors demonstrated a reduction in RPB1 expression (Figure 6E). Consistent with previous studies (37, 49, 51) and our in vitro results (Figure 4), these findings suggest that Minnelide treatment induced RPB1 degradation and inhibition of transcription in human CDS xenograft tumors.

### Minnelide treatment reduces tumor growth in a subset of dFLEx CDS mice.

**Figure 7:**
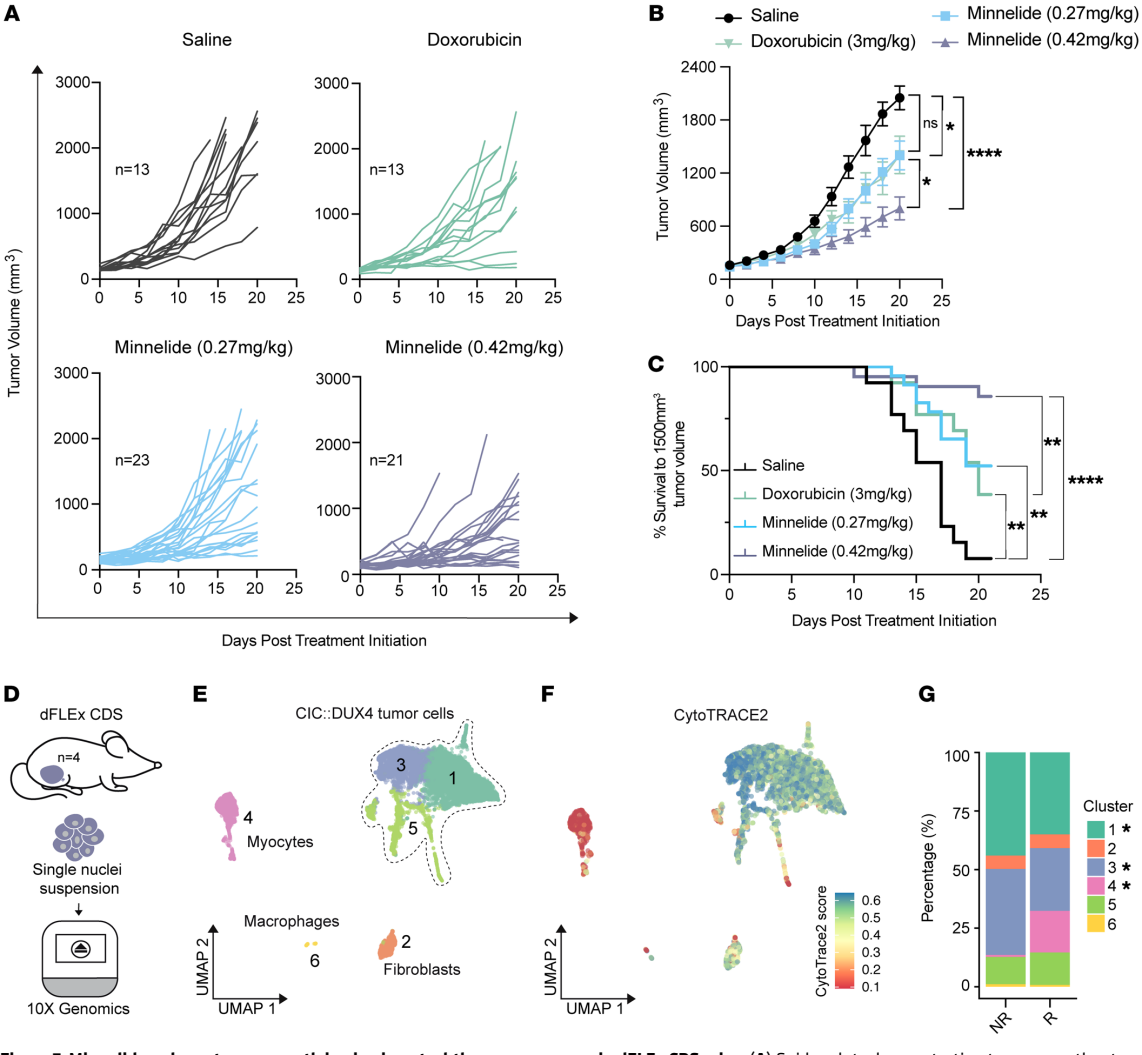
Minnelide reduces tumor growth in vivo in autochthonous sarcomas in dFLEx CDS mice. (**A**) Spider plots demonstrating tumor growth rates in groups treated with control (saline), 3 mg/kg doxorubicin, (0.27 mg/kg), or (0.42 mg/kg) Minnelide over 21 days. (**B**) Comparison of tumor growth across treatment groups. Data are presented as the mean ± SEM. Statistical comparisons were performed at day 21 only. *P < 0.05 and ****P < 0.0001, by 2-way ANOVA. (**C**) Kaplan-Meier curves indicating the time to 1,500 mm^3^ tumor volume. **P < 0.01 and ****P < 0.0001, by log-rank (Mantel-Cox) test. (**D**) Schematic of snRNA-seq workflow. Single-nuclei suspensions for snRNA-seq were prepared from 4 dFLEx CDS tumors treated with 0.42 mg/kg Minnelide for 21 days. (**E**) UMAP plots of tumor and nontumor cells in dFLEx CDS tumors (*n* = 4). Cell types were assigned using the expression of canonical marker genes. (**F**) UMAP of CytoTRACE2 scores. Tumor cluster 3 demonstrated the highest CytoTRACE2 score. (**G**) Cluster composition of dFLEx CDS tumors that demonstrated an unfavorable response (NR) to Minnelide compared with tumors that demonstrated a better response (R) to Minnelide. Overall cluster composition differed significantly between responders and nonresponders (χ^2^ test, *P* = 6.1 × 10^–173^). Three clusters were differentially enriched after FDR correction (denoted by an asterisk): clusters 1 and 3 were enriched in nonresponders (OR = 1.58 and 1.54), while cluster 4 was strongly enriched in responders (OR = 0.035).

Next, to test the efficacy of Minnelide against CDS in an immunocompetent, autochthonous setting, we leveraged our dFLEx CDS model. pCAG-Cre and pCAG-FLPE plasmids were electroporated into the hind limb muscle of dFLEx CDS mice. Once tumors were palpable (~40 days after electroporation), mice were treated daily for 21 days with either 0.42 mg/kg Minnelide, 0.27 mg/kg Minnelide, or vehicle control (saline), or once weekly with 3 mg/kg doxorubicin. Dose selection in this mouse study was informed by previous preclinical studies using Minnelide (37, 42, 61) and the maximum tolerated dose reported in the phase I Minnelide clinical trial for GI cancers (43). A dose of 0.27 mg/kg Minnelide approximates 0.80 mg/m^2^ in patients (60) and has been successfully used in the phase I Minnelide clinical trial for GI cancers (43). Of note, at baseline, tumors from the dFLEx CDS model were very aggressive and grew rapidly. Tumors in the saline control group progressed from palpable to endpoint (~2,000 mm^3^) in 14–20 days (Figure 7, A and B). Treatment with doxorubicin, a backbone therapy in the Ewing chemotherapy regimen, resulted in reduced tumor growth in approximately 23% of mice (3 of 13), while treatment with 0.27 mg/kg and 0.42 mg/kg Minnelide resulted in a marked response in approximately 39% (9 of 23) and approximately 52% (11 of 21) of mice, respectively (Figure 7, A and B) without any appreciable toxicity. Furthermore, treatment with both 0.42 mg/kg and 0.27 mg/kg Minnelide increased the percentage of mice with tumors of less than 1,500 mm^3^ in volume at day 21 (Figure 7C). Collectively these results suggest that the dFLEx CDS model can be leveraged for preclinical testing of therapeutic alternatives for CDS and that Minnelide showed efficacy in multiple CDS preclinical mouse models.

To generate potential hypotheses for why some dFLEx CDS mice respond better to Minnelide than others, we conducted snRNA-seq on dFLEx CDS tumors that demonstrated either a favorable response or a poor response after 0.42 mg/kg Minnelide (Figure 7D and Supplemental Figure 7A). After unsupervised clustering based on the top variably expressed genes, we identified 6 clusters (Figure 7E and Supplemental Figure 7B), 3 of which (clusters 1, 3, and 5) were identified as CIC::DUX4 tumor cells on the basis of their expression of the CIC::DUX4 target genes *Etv1*, *Etv4*, *Etv5*, *Shc3*, *Shc4*, and *Ccnd2* (Supplemental Figure 7C). Using CytoTRACE2 to predict the cellular potency and developmental potential of sequenced cells, we identified tumor cluster 3 as the least differentiated and most stem like (Figure 7F). Interestingly, when we compared the cluster composition of tumors that had a poorer response to 0.42 mg/kg Minnelide (NR) with tumors that had a more favorable response (R), we found that NR tumors had a higher proportion of cluster 3 CIC::DUX4 tumor cells (Figure 7G). These results suggest that a potential mechanism of resistance could be the relative differentiation state of the tumor.

## Discussion

CIC::DUX4 sarcoma is an extremely aggressive cancer driven by expression of a neomorphic transcriptional activator, CIC::DUX4. Although CIC-rearranged sarcomas were recently reclassified by the WHO as a distinct entity from Ewing sarcoma (5), patients are still routinely treated with Ewing-based chemotherapy regimens that have poor efficacy, especially for patients with advanced metastatic disease. A few barriers to identifying better alternative therapeutic options include (a) scarcity of patient samples and patient-derived cell lines and (b) limitations in preclinical models. Although the utilization of patient-derived and ectopically derived xenograft models (12, 13, 15–19) has been critical for our current understanding of CIC::DUX4 biology, these models are limited by the absence of an intact immune system, the bypassing of tumor initiation steps, and the presence of a non-native tumor microenvironment. Here, we aimed to overcome these limitations with the development of a next-generation GEMM of CDS that is both spatially and temporally restricted.

Our previous attempts to generate a conditional CDS GEMM regulated by Cre recombinase were limited because of spontaneous sarcoma development in the absence of Cre recombinase (22). Here, we overcame this problem by using a dual-recombinase FLEx switch strategy that prevented spontaneous tumor formation. The observation that a dFLEx strategy is required to prevent spontaneous CDS tumor formation suggests that frequently used loxP-STOP-loxP cassettes, including those used in our 3 previous CDS GEMMs that spontaneously developed tumors (22), may be insufficient to completely suppress oncogene expression. Taken together, our results support the notion that loxP-STOP-loxP cassettes can, in rare instances, undergo spontaneous deletion or recombination in the absence of Cre recombinase. Whereas expression of an oncogene in a limited number of cells is typically insufficient to initiate tumorigenesis, the exceptional potency of CIC::DUX4 poses an extreme challenge for achieving controlled activation in vivo. This finding has important implications for modeling cancer in mice using loxP-STOP-loxP cassettes. Even when regulating less potent oncogenes or other neoantigens that cannot initiate cancer in the absence of Cre, scientists should be aware that low-level gene expression of a conditional allele in the absence of Cre may occur. In this scenario, expression of the conditional allele may be sufficient to engage immune tolerance mechanisms that might subsequently affect the interaction of the immune system with tumor-initiating cells once Cre is expressed.

To better characterize tumors from the dFLEx CDS model, we conducted snRNA-seq on 4 tumors. Interestingly, while these tumors demonstrated mesenchymal cell–like properties, this model can also generate a sarcoma with striking endothelial cell–like features. Considering that several clinical case reports have described some CIC::DUX4 tumors presenting with endothelial properties (26–29), the dFLEx CDS model appears to have the potential to capture clinically relevant tumor phenotypes. The endothelial cell–like features were present in 1 of 4 tumors analyzed by snRNA-seq (Supplemental Figure 2, A and D). Therefore, further analysis in subsequent studies is needed to characterize the prevalence of the endothelial cell–like variant in the dFLEx CDS GEMM. One possibility for the emergence of transcriptionally distinct CIC::DUX4 tumor cell populations could be tumors arising from different cells of origin. While investigation of the cell of origin for CDS has been limited in part by the lack of suitable model systems, the dFLEx CDS mouse model can be leveraged to investigate this question by crossing this line with mice expressing specific recombinases restricted to specific lineages to further elucidate cell types that are permissive to CIC::DUX4-mediated malignant transformation.

While the dFLEx CDS tumors recapitulate many key histologic features of human CDS, including morphologically small, round-cell tumors with strong expression of CIC::DUX4 (HA tag), ETV4, and WT1, they are not an exact match. In some instances, mouse tumors exhibited potentially unique histologic features, including prominent myxoid stroma and rare foci of chondroid and/or osteoid differentiation (Supplemental Figure 1, B–I). Despite the minor differences between human CDS and the GEMM, the conserved transcriptional program across species suggests that this GEMM closely mimics its human counterpart and can be a useful platform to identify alternative therapeutic approaches for CDS. Moreover, given the precedent for rare osteoid differentiation in other fusion-associated sarcomas, such as synovial sarcoma (62), BCL-6 corepressor–associated (BCOR-associated) sarcoma (63), and Ewing sarcoma (64), and the rarity of CDS, the possibility that this may be recognized clinically in the future cannot be entirely excluded.

In addition to developing the dFLEx CDS mouse model, we also conducted a small-molecule library screen to identify Minnelide, an inhibitor of RNAP II–mediated transcription, as a potential therapeutic approach for CDS. Using an array of in vitro approaches, we found that Minnelide’s mechanism of action in CDS cells was mediated through targeting of XPB, which stalled RNAP II at gene promoters. Prolonged stalling of RNAP II led to hyperphosphorylation of RPB1 (the largest subunit of RNAP II) at Ser5 and then, subsequently, ubiquitination and proteasome-mediated degradation of RPB1, leading to apoptosis.

Interestingly, several mechanisms have been proposed for how RPB1 loss leads to cell death (37, 57, 58). Given that CDS is driven by the activation of a unique oncogenic transcriptional program, we anticipated that Minnelide would suppress this CIC::DUX4-specific transcriptional program, resulting in loss of essential gene expression after mRNA decay to induce cell death. Unexpectedly, there were few CIC::DUX4 target genes whose expression decreased 2–24 hours following Minnelide treatment. Instead, we found that the initiation of cell death closely coincided with the loss of RPB1 expression. These results align with recent findings that RNAP II degradation in the absence of mRNA decay is sufficient to trigger the intrinsic pathway of apoptosis (58). While it is still possible that loss of expression of the CIC::DUX4 target genes *DUSP4*, *VGF*, and *MYC* at 24 hours contributed to Minnelide-mediated cell death, a transcription-independent mechanism of cell death in CDS would provide a rationale for the specificity of Minnelide in CDS cells over normal adult tissues, whose mitochondria are more refractory to proapoptotic signaling (65). Although a previous study reported that degradation of RNAP II shifted the localization of BCL2L12 from the nucleus to mitochondria via a PTBP1 mechanism to trigger the intrinsic pathway of apoptosis (58), we were not able to detect PTBP1 translocation in Minnelide-treated human CDS cells. Therefore, alternative pathways exist to mediate the Poll II degradation–dependent apoptotic response in CDS.

As there is an urgent unmet clinical need for alternative therapeutic strategies for CDS, we used 2 different murine models to test the efficacy of Minnelide against CDS in vivo. We started by inducing tumors in the dFLEx CDS model, and then treated mice daily for 21 days with either 0.42 mg/kg Minnelide, 0.27 mg/kg Minnelide, or saline, or once weekly with 3 mg/kg doxorubicin. Interestingly, at 0.27 mg/kg Minnelide, we noted a split response (responders/ nonresponders) in the dFLEx CDS model. In contrast, the human CDS xenograft model demonstrated reduced tumor growth in almost every mouse tested. These results not only highlight the value of the dFLEx CDS model, but also emphasize the importance of utilizing multiple preclinical models when testing potential therapies for CDS. Given that Minnelide does not directly target the CIC::DUX4 fusion or mechanisms specific to CDS, future studies identifying therapeutic agents specific to CDS are still needed. Importantly, 2 recent studies utilizing CDS tumoroids identified myeloid cell leukemia-1 (MCL-1) inhibitors as a promising therapeutic approach for CDS (66, 67). Future studies could test MCL-1 inhibitors and other therapeutics identified in the tumoroid models in the immunocompetent dFLEx CDS mouse model.

## Methods

### Sex as a biological variable.

All in vivo studies included both male and female animals, and similar results were observed in both sexes.

### Tumor induction in dFLEx CDS mice.

Six- to 12-week-old dFLEx CDS mice were anesthetized, and then 30 μg naked pCAG-Cre (Addgene plasmid 13775) and pCAG-FLPE (Addgene plasmid 13787) plasmid DNA diluted in sterile saline was injected into the gastrocnemius using a 31 gauge insulin syringe. As previously described (68), needle electrodes with a 5 mm gap were inserted into the muscle to encompass the DNA injection site, and electric pulses were delivered using an electric pulse generator (Electro Square Porator ECM830, BTX). Three 100 V pulses followed by 3 additional 100 V pulses of the opposite polarity were administered to each injection site at a rate of 1 pulse per 50 ms, with each pulse being 200 ms in duration. Palpable tumors were detected starting at post-electroporation day 30. Metadata for dFLEx CDS mice used in this study can be found in Supplemental Table 2. pCAG-Cre and pCAG- FLPE were a gifts from Connie Cepko (Addgene plasmids 13775 and 13787, Harvard University, Cambridge, Massachusetts, USA) (69).

### IHC.

As previously described (22) tissue samples were fixed in 10% formalin/70% EtOH, then embedded in paraffin blocks. Sections (0.4 μM) ere mounted and stained with H&E or antibodies. Detection of antibodies was performed using the Vectastain Elite ABC-HRP Kit (Vector Laboratories, VECTPK6100) and the DAB Peroxidase Substrate Kit (Vector Laboratories, VECTSK4100). Antigen unmasking was performed with a citrate buffer, pH 6.0, by a modified microwave retrieval method followed by boiling. Images were captured on an Aperio AT2 bright-field scanner (Leica Biosystems) using the UPlanS Apo 20×/0.75 NA (high-resolution ×20 objective). The following antibodies were used for IHC analysis: HA (Cell Signaling Technology, 3724) at 1:800; ETV4 (Proteintech 10684-1-AP) at 1:1,800; CD99 (Thermo Fisher Scientific, MA5-12287) at 1:200; WT1 (Thermo Fisher Scientific, MA5-32215) at 1:2,000; pan-cytokeratin (Abcam, ab9377) at 1:200; desmin (Abcam, ab15200) at 1:200; and p-RPB1 CTD (Ser2/Ser5) (Cell Signaling Technology, 13546) at 1:100.

### Cell viability drug screen.

The Tocriscreen Epigenetics 3.0 compound library (catalog 7578) was used in the cell viability drug screen. Kitra-SRS, CDS#2, and X1C1 CDS cell lines (see Supplemental Methods) were plated at a density of 3 × 10^4^ to 5 × 10^4^ cells per well in 96-well plates. In technical triplicate, cells were incubated in drug at 4 different concentrations (1, 10, 100, and 1,000 nM) for 72 hours and then assayed using CellTiter-Glo luminescence (Promega, G7572) to assess cell viability. The plates were read using a CLARIOstar Plus plate reader, and RLU was normalized to DMSO-treated cells. For visualization, 20 compounds with the largest overall effect size and variance were selected. Compounds were ordered by drug class, and the percentage of viability across conditions was plotted as a heatmap using the pheatmap package in R.

### Annexin V/PI flow cytometry.

The Dead Cell Apoptosis Kit with Annexin V for Flow Cytometry (Thermo Fisher Scientific, V13245) was used to detect apoptotic cells. CDS cells were trypsinized and then pelleted by centrifugation at 300*g* for 3 minutes. Cell pellets were resuspended in 1× annexin-binding buffer, passed through a 70 μm cell strainer, and then transferred to Falcon round-bottomed polystyrene test tubes equipped with 35 μm nylon mesh caps. Cell suspensions were adjusted to a density of 1.0 × 10^6^ cells/mL, and then Alexa Fluor 488, annexin V, and PI were added according to the manufacturer’s instructions. Cells were incubated for 15 minutes at RT and then analyzed on a BD LSR Fortessa X20 flow cytometer.

### Immunofluorescence.

Cells were grown on 12 mm poly-l-lysine–coated glass coverslips (Neuvitro, catalog GG-12-15-PLL) and then washed with Dulbecco′s Phosphate Buffered Saline with MgCl_2_ and CaCl_2_ (MilliporeSigma, D8662) for 5 minutes at 37°C, prior to fixation in 4% paraformaldehyde (PFA) (Electron Microscopy Sciences, catalog 15710) for 10 minutes at RT. Coverslips were incubated in blocking buffer (0.2% Triton X-100, 2.5% BSA) for 1 hour at RT, then in either CC3 (Cell Signaling Technology, 9661, 1:400), PTBP1 (Cell Signaling Technology, 57246, 1:500), or gH2A.X (Cell Signaling Technology, 2577, 1:400) overnight at 4°C. Next, coverslips were incubated with donkey anti-rabbit secondary antibody for 1 hour at RT (Alexa Fluor 488 or Alexa Fluor 555, 1:1,000, Invitrogen, Thermo Fisher Scientific, catalog A-11001, A-32794). For the PTBP1 experiments, following incubation with a secondary antibody, the coverslips were incubated with CoraLite Plus 488–Phalloidin (Proteintech, PF00001, 1:400) for 20 minutes at RT. Coverslips were mounted on Superfrost slides (Thermo Fisher Scientific, catalog 22-037-246) with ProLong Gold Anti-Fade Mountant with DAPI DNA Stain (Invitrogen, Thermo Fisher Scientific, catalog P36931). Images were obtained on a Leica Stellaris confocal microscope equipped with an HC PL APO HCX PL APO ×40/0.95 NA CORR (0.11–0.23 mm coverslip correction) lens, using a Hamamatsu Camera, and then processed using Fiji software.

### DNA fiber assay.

A DNA fiber assay was performed as described previously (70). Briefly, cells were plated 24 hours prior to treatment. Cells were incubated in Minnelide (MedChemExpress, HY-124584, dissolved in H_2_O) at 25 nM for 48 hours. For the measurement of replication rates, cells were sequentially labeled with 25 μM 5-chloro-2′-deoxyuridine (CldU) (MilliporeSigma, C6891-100MG) for 20 minutes, and then 250 μM 5-iodo-2′-deoxyuridine (IdU) (MilliporeSigma I7125-5G) for 20 minutes. Cells were harvested and diluted to a concentration of 5 × 10^5^ cells/mL in PBS. Cell suspension (3 μL) was mixed with 7 μL lysis/spreading buffer (0.2 M Tris-HCl, pH 7.4, 40 mM EDTA, 0.5% SDS) on a microscope slide (Superfrost Plus, Fisherbrand, Thermo Fisher Scientific) and tilted to allow DNA fiber spreading. Slides were then air-dried, and fibers were fixed in 3:1 methanol/acetic acid for 10 minutes. DNA was denatured in 2.5 M HCl for 1 hour, washed in 1× PBS, and then incubated in blocking solution (1% BSA, 0.1% Tween-20 in PBS) for 1 hour. The slides were then incubated in monoclonal rat anti-BrdU (BU1/75 [ICR1], Abcam, ab6326, 1:1,000) and mouse monoclonal anti-BrdU (clone B44, BD, 347580 [7580], 1:1,000) overnight at 4°C. Slides were fixed in 4% PFA for 10 minutes and incubated in goat anti–rat Alexa Fluor 555 (Cell Signaling Technology, 4417) and goat anti–mouse Alexa Fluor 488 secondary antibodies (Thermo Fisher Scientific, A-10680, 1:1,000) for 45 minutes at RT. DNA fibers were visualized at ×63 magnification using a confocal microscope. The lengths of CldU and IdU tracks were measured using Fiji software and converted into kb pairs using the conversion factor 1 μm = 2.59 kb. At least 100 forks were analyzed for every condition.

### FLICK assay.

The FLICK assay was performed as previously described (59). Briefly, cells were seeded at a density of 6,000 cells per well in 90 μL culture media in 2 separate 96-well plates (Thermo Fisher Scientific, 165305), 24 hours prior to the start of the assay. One plate was designated as the experimental (test) plate, and the second plate was used to establish baseline (T0) values. On the following day, cells were treated with either 25 nM (Kitra-SRS, ECD1 and CDS#2) or 70 nM (X1C1) Minnelide (MedChemExpress, HY-124584, dissolved in H_2_O). Simultaneously, 3 μM SYTOX Green (Thermo Fisher Scientific, S7020) was added to all wells of both the test and T0 plates. The test plate was sealed with a sterile film and immediately imaged on a CLARIOstar Plus plate reader at an excitation/emission of 504/523 and a gain of 700. Following imaging, the film was removed, and the test plate was returned to the incubator. Imaging of the test plate was repeated at multiple time points over a 72-hour Minnelide treatment period. To determine the total fluorescence (TF) of the test plate, 1.0% Triton-X was added to all wells after the final time point, followed by incubation at 37°C with 5% CO_2_ for 2 hours. The test plate was then reimaged using the same settings. To establish the TF at the start of the assay (T0), immediately following SYTOX Green addition on day 0, Triton X-100 (1.0%) was added to all wells of the T0 plate. After a 2-hour incubation (37°C, 5% CO_2_) to ensure complete lysis, the plate was imaged as described above. The lethal fraction and onset of cell death were calculated using the methods described previously (59).

### EU incorporation.

EU incorporation assays were conducted as described previously (58). Briefly, cells treated with 25 nM (Kitra-SRS, ECD1 and CDS#2) or 70 nM (X1C1) Minnelide (MedChemExpress, HY-124584, dissolved in H_2_O) were labeled with 1 mM EU for 1 hour in existing media. Cells were then washed with PBS, trypsinized, pelleted, and washed with cold PBS. Cells were then fixed in 4% fresh paraformaldehyde in PBS at RT for 15 minutes. Fixed cells were washed with cold PBS, pelleted, resuspended in ice-cold 100% methanol, and then stored at –20°C overnight. The following day, methanol was removed, and cells were washed with PBS plus 0.1% Tween-20. Cells were then incubated for 30 minutes at RT (protected from light) with 100 μL Click-iT reaction master mix as per the manufacturer’s instructions (Thermo Fisher Scientific, C10329). Cells were washed once with 100 μL Click-iT reaction rinse buffer (Thermo Fisher Scientific, C10329) and then washed with PBS plus 0.1% Tween-20. Samples were resuspended in PBS plus 0.1% Tween-20, filtered, and analyzed on a BD LSR Fortessa X20 flow cytometer. Data analysis was conducted using FlowJo software.

### Xenograft tumor formation and evaluation of Minnelide in vivo.

Six- to 8-week-old NOD-SCID IL2Rγ-null mice (The Jackson Laboratory, 005557) were s.c. transplanted with 1.0 × 10^7^ Kitra-SRS or ECD1 cells resuspended in a 100 μL, 1:1 mixture of culture media and Matrigel (Corning, 356231). Palpable tumors at the site of injection were detected starting on post-transplantation day 7. Beginning at the time of tumor palpation [~200–300 mm^3^ by digital caliper measurement using the formula (length × width^2^)/2], mice were randomized into 1 of 3 groups: (a) 0.21 mg/kg Minnelide (MedChemExpress, HY-124584), (b) 0.27 mg/kg Minnelide, or (c) saline. Minnelide was initially dissolved in H_2_O and then further diluted in saline. Each mouse received 0.21 mg/kg Minnelide, 0.27 mg/kg Minnelide, or saline by daily i.p. injection for 21 consecutive days. Tumor volume was measured every 2–3 days by digital caliper, and tumor volume was calculated using the formula (length × width^2^)/2. Because individual mice reached palpable tumor volumes (~200–300 mm^3^) at different time points, tumor measurements obtained within each 3-day interval were averaged and assigned to the midpoint of that interval (e.g., day 2), allowing consistent comparison of growth kinetics across animals.

### Evaluation of Minnelide in dFLEx CDS mice.

For the evaluation of Minnelide in the dFLEx CDS mouse model, tumors were induced using the methods described previously. Mice were treated daily for 21 consecutive days with 0.42 mg/kg Minnelide (MedChemExpress, HY-124584), 0.27 mg/kg Minnelide, or saline, or they were treated once weekly with 3 mg/kg doxorubicin by i.p. injection, beginning at the time of tumor palpation (~30–70 days following electroporation). Tumors were measured every 2 days by digital caliper, and the tumor volume was calculated using the formula (length × width^2^)/2. Mice were monitored until they reached the approved tumor burden limit of 2,000 mm^3^. For survival analysis, the time to a tumor burden of 1,500 mm^3^ was used as a standardized measure of tumor progression, as tumors frequently exceeded 2,000 mm^3^ between measurement intervals, making the precise time of reaching this limit difficult to determine. pCAG-Cre and pCAG- FLPE were a gifts from Connie Cepko (Addgene plasmid nos. 13775 and 13787, Harvard University, Cambridge, Massachusetts, USA) (69). Control sarcomas from KRAS^G12D^ p53^fl/fl^ (KP) mice were generated as previously described (71).

### Statistics.

Results are presented as the mean ± SEM unless otherwise indicated. Prior to statistical analysis, all datasets were displayed graphically (box-and-whisker plots using GraphPad Prism 10) and assessed for normality using the Shapiro-Wilk test to determine whether parametric or nonparametric tests should be used. One-way ANOVA (at day 21) or 2-way ANOVA was used to compare tumor growth between the Minnelide- and saline-treated groups. Survival data were analyzed using Kaplan-Meier curves with the log-rank test for statistical significance. All calculations were performed using GraphPad Prism 10 (GraphPad Software). Additional details on each statistical test used can be found in the Supporting Data Values file. A *P* value of less than 0.05 was considered statistically significant.

### Study approval.

All animal experiments were approved by the Duke University Animal Care and Use Committee (protocol no. A014-22-01) and the University Health Network’s Princess Margaret Cancer Centre Animal Care Committee and aligned with guidelines from the Canadian Council on Animal Care (protocol no. 6825).

### Data availability.

All RNA-seq data are freely accessible through the Gene Expression Omnibus (GEO) database (GEO GSE328405, GSE328406, and GSE328407). The dFLEx CDS mouse line is available at The Jackson Laboratory (stock no. 041280). Values for all data points in graphs are reported in the Supporting Data Values file. Code used for data analysis is available upon request. Additional information on methods used for this study can be found in Supplemental Methods.

## Author contributions

This project was conceptualized and designed by PGH, DGK, and MRB with support from KMO and JEH. All experiments were performed by MRB with support from PGH, AVS, and RB. Animal breeding and maintenance were performed by BA. Histological analysis was conducted by BCD. This manuscript was written by MRB, with editing by PGH and DGK. All authors approved the submission of this manuscript.

## Conflict of interest

DGK is a member of the scientific advisory board and owns stock in Lumicell Inc., a company commercializing intraoperative imaging technology. This affiliation does not represents a conflict of interest with respect to the work described in this manuscript. DGK is a coinventor on a patent for a hand-held imaging device and is a coinventor on a patent for radiosensitizers (imaging device: “System and method for large field of view, single cell analysis” [patent US-11730371-B2]; radiosensitizer: “Dual atm and dna-pk inhibitors for use in anti-tumor therapy” [US-20220142995-A1]). Merck has provided research support to DGK in the past.

## Funding support

This work is the result of NIH funding, in part, and is subject to the NIH Public Access Policy. Through acceptance of this federal funding, the NIH has been given a right to make the work publicly available in PubMed Central.

- National Cancer Institute grant 7R35CA197616 (to DGK).
- Department of Defense grant W81XWH-22-1-0454 (to DGK).
- Conquer Cancer, The ASCO Foundation and QuadW Foundation Young Investigator Award 2024YIA-7001324982 (to PGH).
- Alex’s Lemonade Stand Foundation grant 1442898 (to PGH).
- National Institute of Child Health and Human Development T32 grant T32HD040372, awarded to the Developmental and Stem Cell Biology program at Duke University (to MRB).

## Supplementary Material

Supplemental data

Unedited blot and gel images

Supplemental table 1

Supplemental table 2

Supplemental table 3

Supporting data values

## Version 1 06/16/2026

In-Press Preview

## Version 2 07/15/2026

Electronic publication
