# Supplementary material for: Targeting CIC::DUX4 sarcoma with Minnelide in a dual recombinase–initiated genetically engineered mouse model
Source: J Clin Invest. 2026 Jun 16;136(14):e202218. doi: 10.1172/JCI202218 (PMC13367964; doi:10.1172/JCI202218)
Supplement: Supplemental data [file jci-136-202218-s275.pdf]

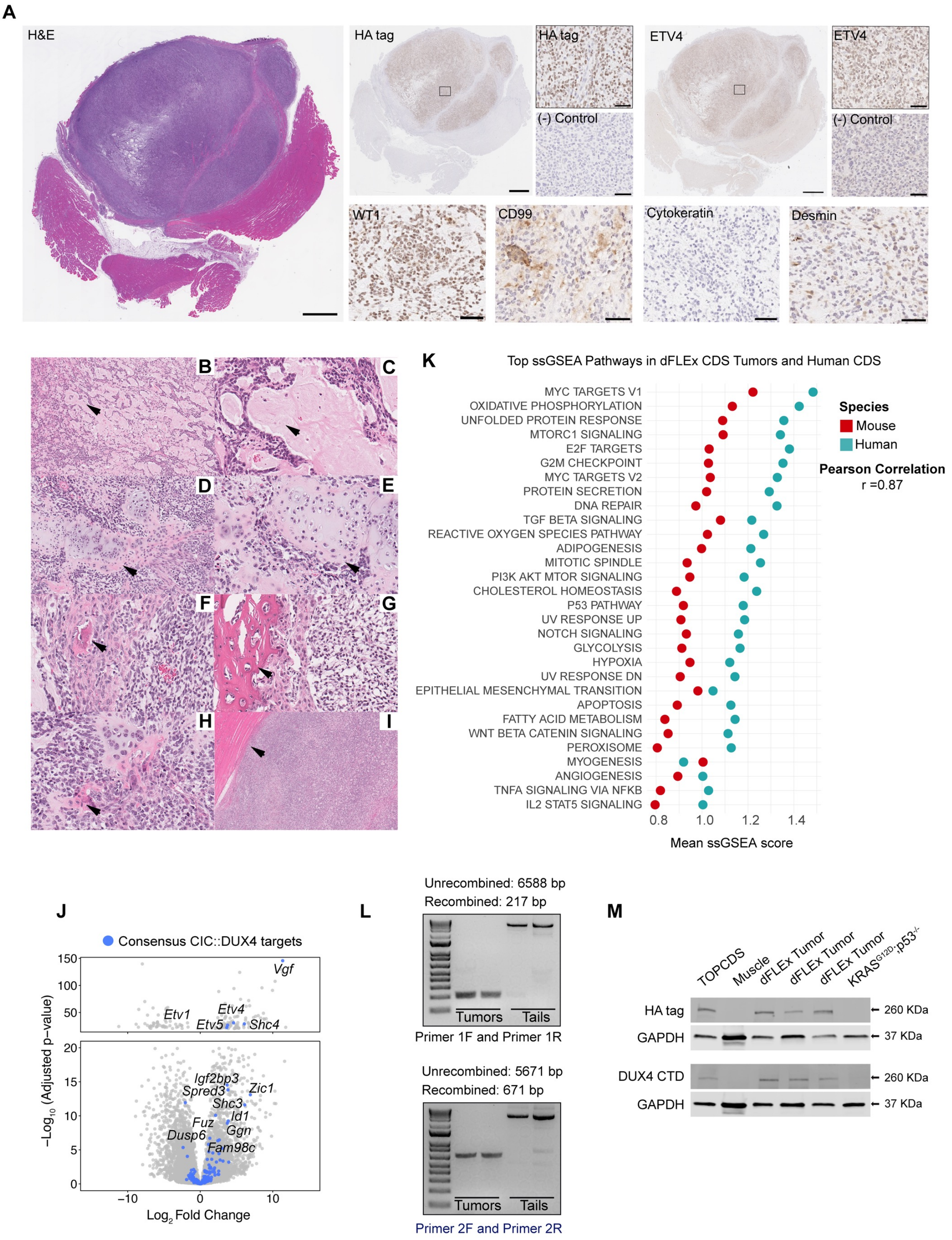

Supplementary Figure 1. A. Immunohistochemistry on tumors formed in dFLEx CDS mice after electroporation of Cre + FLPE plasmids. Sarcomas from *$KRAS^{G12D}$; $Trp53^{fl/fl}$* (KP) mice used as negative (-) control. Scale bars 1500 µm (entire tumor sections) and 50µm (insets denoted by boxes). **B-I.** Representative photomicrographs from the mouse model highlighting histologic features less characteristic of human CIC::DUX4 sarcomas (**B&C**) Prominent myxoid stroma (arrow) (100x and 400x). (**D&E**) Rare foci of chondroid differentiation showing chondrocytes with mild atypia within lacunae in a hyaline chondroid matrix (arrows; 200× and 400×). (**F&G**) Rare foci of osteoid differentiation showing osteocytes with mild atypia within lacunae in an osteoid matrix (arrows; 200× and 400×). (**H**) Nodule with chondroid differentiation undergoing endochondral-type ossification, with focal matrix mineralization (arrow) and osteoclast-type giant cells (400x). (**I**) Circumscribed tumor margin (arrow), in contrast to the infiltrative margins typically seen in human tumors (40x). **J**. Volcano plot demonstrating induction of CIC::DUX4 transcriptional target genes in dFLEx CDS tumors relative to KP control. **K.** GSEA pathway analysis on dFLEx CDS tumors compared to human CDS cell lines **L.** DNA gel of amplification products from PCR of genomic DNA from tumors and tails using primers indicated in the schematic in Figure 1A that span the loxP and FRT sites. In the tumors a ~217bp and ~671bp product is amplified consistent with recombination. **M.** Western blot on lysates from dFLEx CDS tumors probed for the HA-tag and DUX4 CTD confirming the expression of a ~ 260kD protein in dFLEx CDS tumors (n=3). TOPCDS mouse CDS cell line (Hendrickson et al. Oncogene 2024) was used as a positive control and dFLEx CDS contralateral muscle and a KP cell line were used as negative controls.

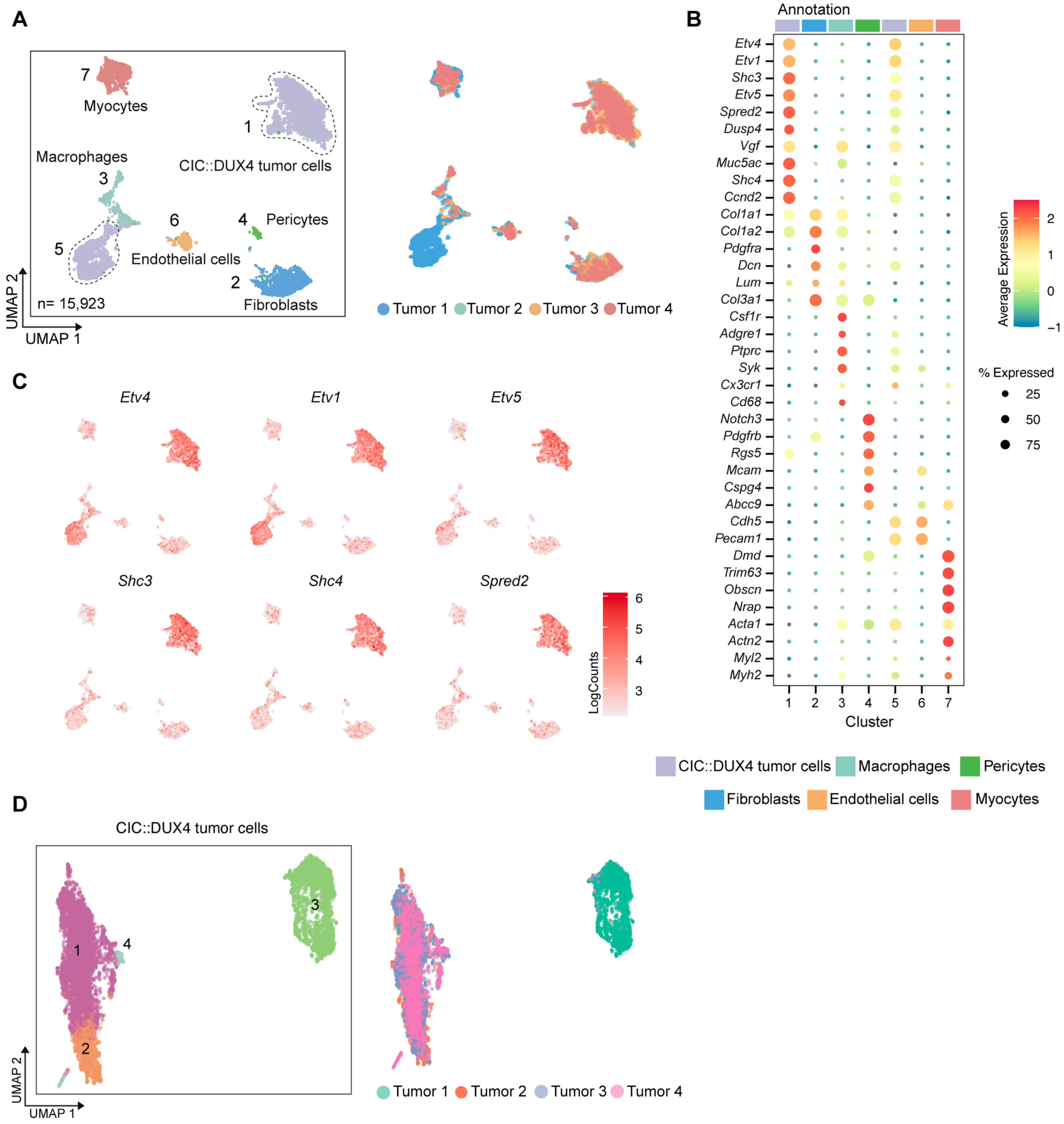

Supplementary Figure 2. A. UMAP of single cells colored by sample of origin. **B.** Dot plot highlighting marker gene expression in tumor clusters **C.** UMAPs highlighting the expression of CIC::DUX4 target genes *Etv1, Etv4, Etv5, Shc3, Shc4* and *Spred2*. **D.** UMAP of tumor cells colored by sample of origin.

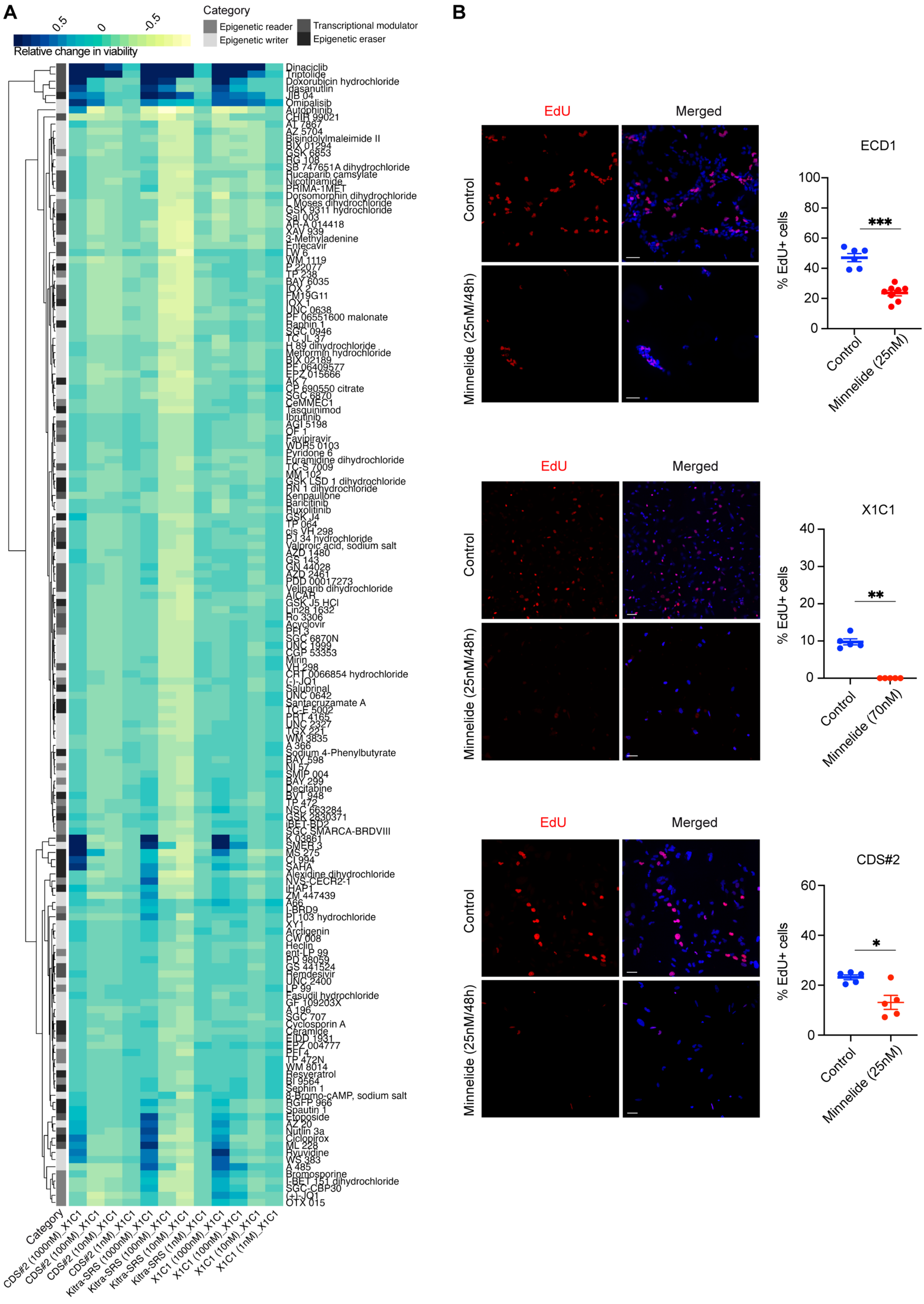

Supplementary Figure 3. A. Viability drug screen on human CDS cells using the Tocriscreen Epigenetics 3.0 compound library. Top 100 compounds at 1nM, 10nM, 100nM and 1000nM concentrations. **B.** EdU labeling on human ECD1, X1C1 and CDS#2 CDS cells treated with Minnelide for 48h. Statistical comparisons were performed using a two-tailed exact Mann-Whitney test. A total of 5-8 regions per condition were quantified, including regions sampled from 3 independent experiments. *p<0.05, **p<0.01, ***p<0.001 (scale bars 50µm).

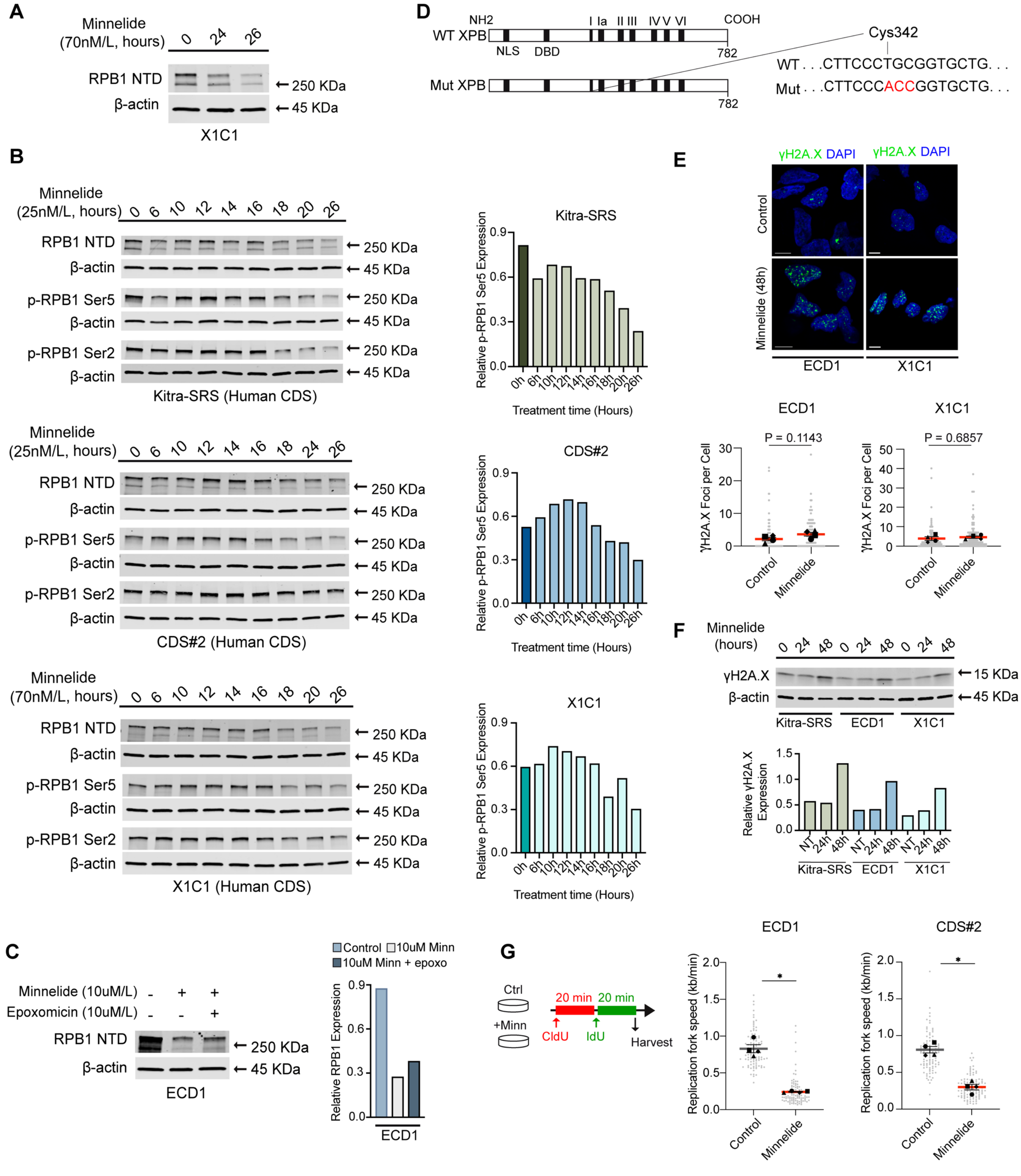

Supplementary Figure 4. A. Western blot of RPB1 expression in human X1C1 cells after treatment with 70nM Minnelide for 24 and 48h. **B.** Western blot of RPB1 expression and phosphorylated RPB1 over a time course of Minnelide treatment in human Kitra-SRS, CDS#2, and X1C1 cells. Quantification demonstrates transient increase of p-RPB1 Ser5 expression in all cell lines. **C.** Western blot demonstrating proteosome inhibitor epoxomicin partially rescues Minnelide mediated RPB1 degradation in human ECD1 cells. **D.** Strategy for generating a Minnelide-resistant XPB mutant. **E.** gH2A.X immunofluorescence on human ECD1 and X1C1 cells treated with Minnelide for 48h (scale bars 10μm). Statistical comparisons were performed using a two-tailed exact Mann-Whitney test. At least 50 nuclei per condition were quantified, including nuclei sampled from 4 independent experiments. **F.** Western blot of gH2A.X in human Kitra-SRS, ECD1 and X1C1 cells, 24 and 48h after Minnelide treatment (top) with quantification (bottom). **G.** DNA fiber assay on human ECD1 and CDS#2 and cells treated with Minnelide for 48h. Statistical comparisons were performed using a two-tailed exact Mann-Whitney test. At least 100 fibers per condition were quantified, including fibers sampled from 4 independent experiments. *p<0.05

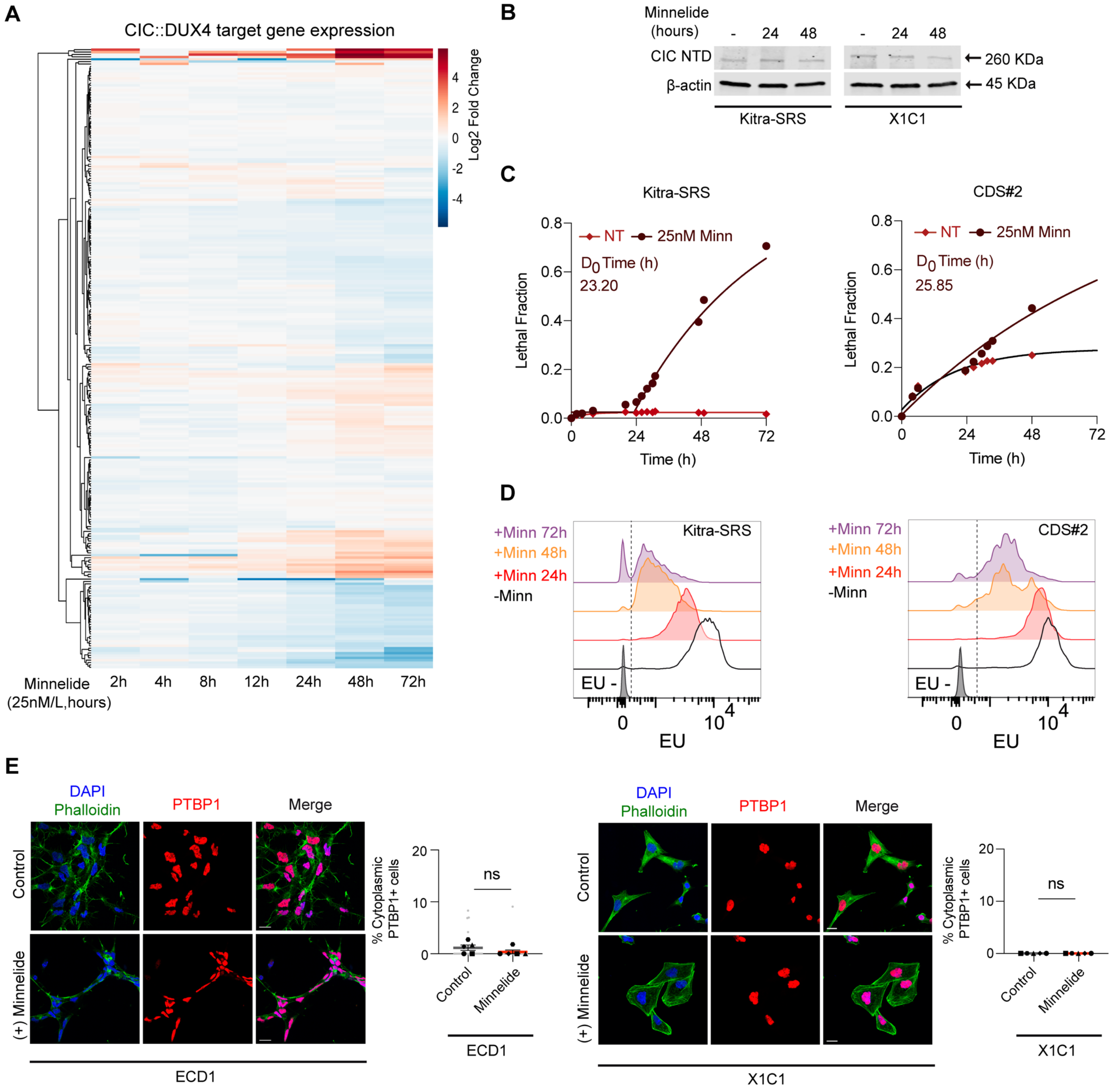

Supplementary Figure 5. A. Heatmap showing expression of CIC::DUX4 target genes following Minnelide treatment for 2, 4, 8, 12, 24, 48, and 72 hours. The majority of CDS target genes, including *ETV1/4/5*, remained largely unchanged until 72 hours post-treatment. **B.** Western blot of CIC::DUX4 expression in human Kitra-SRS and X1C1 cells 24 and 48h after Minnelide treatment. **C.** FLICK assays on human Kitra-SRS and CDS#2 cells demonstrating the onset of cell death is 23.20h and 25.85h after the start of Minnelide, respectively. **E.** EU incorporation 24, 48 and 72h after Minnelide treatment to assess changes in nascent transcription in Kitra-SRS and CDS#2 cells **E.** PTBP1 immunofluorescence on human ECD1 and X1C1 cells after 48h Minnelide treatment (scale bars 20µm). Statistical comparisons were performed using a two-tailed exact Mann-Whitney test. A total of 24-25 regions per condition were quantified, including regions sampled from 5 independent experiments.

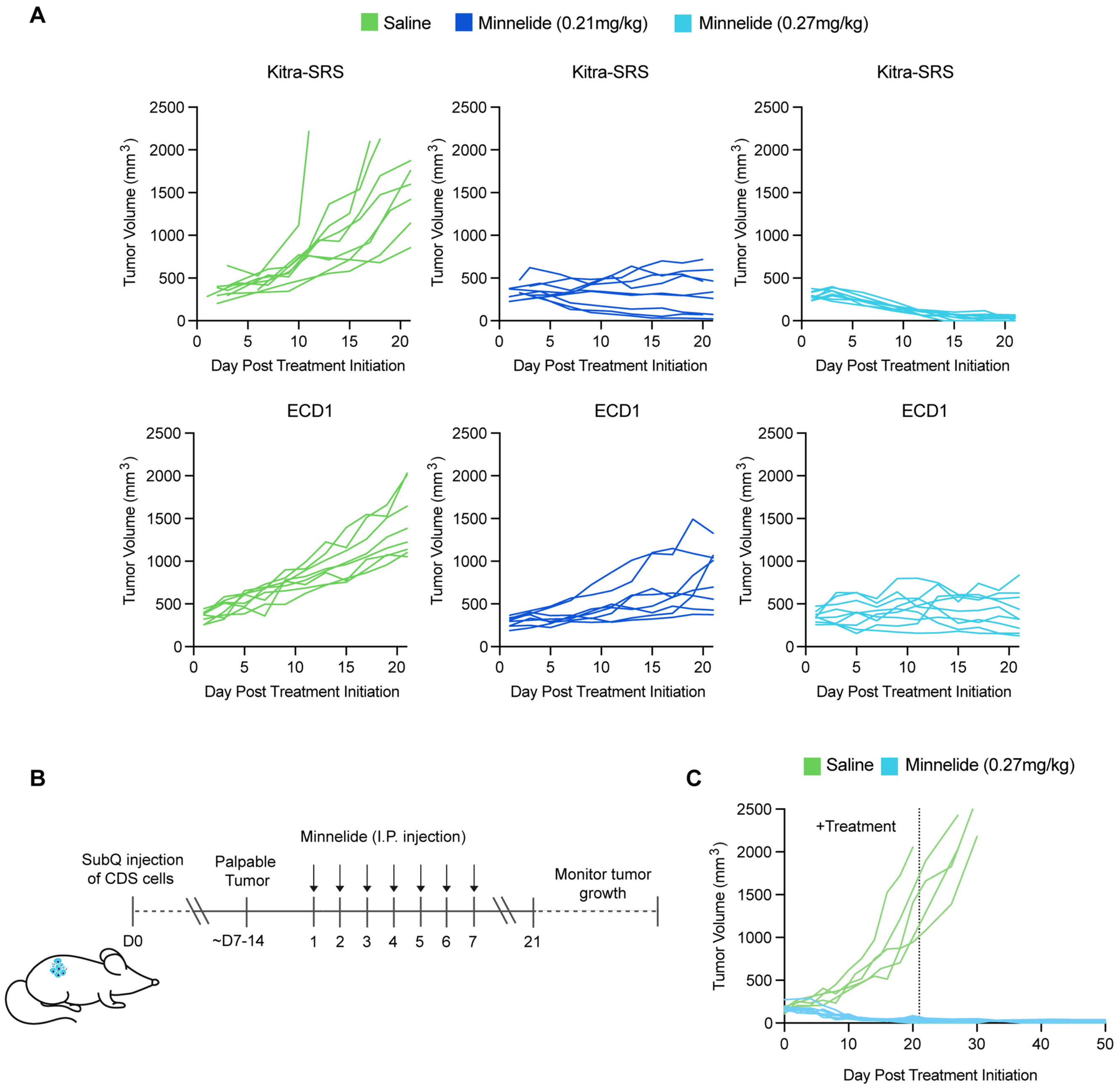

Supplementary Figure 6. A. Spider plots demonstrating tumor growth rates in xenograft mice shown in Figure 6B. **B.** Schematic outlining the workflow for assessing durability of response in the xenograft models. **C.** Spider plots demonstrating tumor growth of control (saline) treated mice and mice treated with 0.27mg/kg Minnelide for 21 days. Mice were followed out to 50 days to assess durability of response.

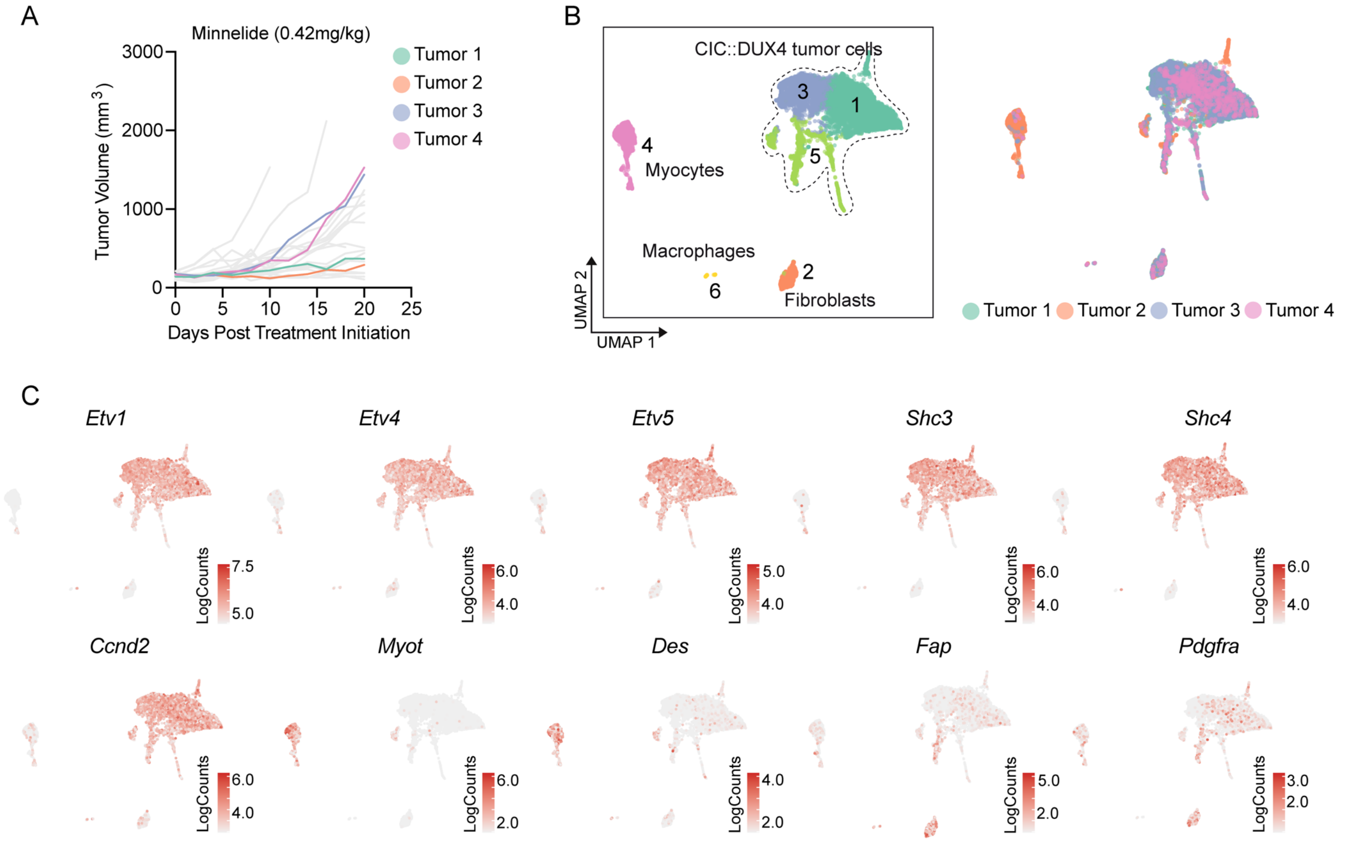

Supplementary Figure 7. A. Spider plots demonstrating tumor growth of mice selected for snRNAseq studies **B.** UMAP of single cells colored by sample of origin. **C.** UMAPs demonstrating the expression of marker genes that helped inform cell type annotation

Supplementary methods

Generation of Transgenic Animals

dFLEx CDS mice were generated by Ozgene (Perth, WA, Australia). An inverted 3xHA CIC exon and inverted CIC/DUX4-CTD exon (inverted 3xHA_CIC exon_lox2272_loxP_FRT'_FRT_inverted CTC_DUX4-CTD exon_FRT'_SV40pA_KT3_FRT_DUX4-CTS exon_SP6 promoter_BGHpA) was cloned into a Rosa26 targeting construct. After sequence verification, the construct was electroporated into ES cells and selected in antibiotic (neomycin)-containing media. Clones containing the knock-in were screened and validated by qPCR, then injected into goGermline blastocysts, which were transplanted into female nurse mice for gestation and delivery of goGermline heterozygous males. GoGermline heterozygous male mice were then bred to generate 100% ES cell-derived dFLEx CDS mice.

Genotyping

Genomic DNA (gDNA) was purified from tail clips using the Qiagen DNeasy Blood and Tissue kit (Cat. 69504). Primers designed to amplify across a region of the stop cassette into the inverted exon 1 (Supplementary Table 1) were used to validate the presence of the dFLEx CDS allele. PCR was performed using 2X Taq FroggaMix (Cat. FBTAQM) and optimized for amplicon size. To validate recombination, gDNA was purified from dFLEx CDS tumors, and primers were designed to amplify across the LoxP and FRT sites (Supplementary Table 1). PCR on dFLEx CDS tumors was performed using 2X Taq FroggaMix. As a control, PCR utilizing the same primers was conducted on tail clips from dFLEx CDS mice. To accommodate the

unrecombined large amplicon size, PCR was performed using NEB LongAmp Taq DNA Polymerase (Cat. M0323S).

RNA Sequencing

RNA was extracted and purified from flash-frozen tumors and cells using a Qiagen RNeasy Plus kit (Cat. 74134). As described previously (22), high-quality RNA (RIN >7) was divided into duplicates, from which 150bp paired-end, rRNA-depleted libraries were made using the Illumina TruSeq RNA Library Prep Kit (Illumina, CA, USA). Libraries were quantified using the KAPA Library Quantification kit (KAPA Biosystems, MA, USA), multiplexed, clustered onto flowcells, and then sequenced using an Illumina HiSeq 4000 sequencer (or equivalent platform) by GENEWIZ (Azenta, NJ, USA). Raw sequencing reads were trimmed using Trimmomatic v0.39 (ILLUMINACLIP:TruSeq3-PE-2.fa:2:30:10:2:keepBothReads LEADING:3 TRAILING:3 MINLEN:36).

RNA Sequencing Analysis

To enable quantification of the *CIC::DUX4* transcript, the mm39 reference genome FASTA was appended to include the *HA-CIC::DUX4* sequence. Additionally, the corresponding gene annotation file was modified to include features (*Cic*, *DUX4* exons, and HA-tag). Trimmed sequencing reads were then aligned to the appended mm39 reference genome (dFLEx) or hs38 using default parameters in STAR v2.7.10a. FeatureCounts (Subread v2.0.3) was used to compile a count table from sorted and indexed BAM files, which was loaded into DESeq2 to calculate differential expression. Volcano plots were generated using ggplot2, with statistical significance

defined as padj < 0.05. Genes with padj < 0.05 and log2FC > 0 were classified as "upregulated," padj < 0.05 and log2FC < 0 as "downregulated," and all others as "not significant." For Supplementary Figure 1J, the top 14 most significantly upregulated CIC::DUX4 target genes were selected for labeling. Mean expression values for all 235 CIC::DUX4 target genes can be found in Supplementary Table 3.

Transcriptional comparison of dFLEx CDS tumors and human cell lines

Gene expression and metadata for fusion-positive sarcoma cell lines were obtained from the publicly available DepMap dataset. All CIC::DUX4 sarcoma (CDS), undifferentiated pleomorphic sarcoma (UPS/MFH), Ewing sarcoma (ES), and alveolar rhabdomyosarcoma (ARMS) cell lines available in DepMap were included in this analysis. Mouse tumor (dFLEx CDS and KP) RNA-seq data were processed as described in the RNAseq methods section. To enable cross-species comparison, orthologous gene mappings between human and mouse were obtained from the Mouse Genome Informatics (MGI) homology database, and one-to-one orthologs were identified by selecting gene pairs with unique mappings between species. To define a CDS gene signature, genes were ranked based on log2 fold-change between human CDS and UPS cell lines. UPS was selected as the comparison group to align with analyses comparing dFLEx CDS tumors to KP tumors. The top 100 upregulated and top 100 downregulated genes were selected and combined to generate a 200-gene CDS signature, then gene expression values were z-score normalized across samples for each gene independently within each species. Pearson correlation coefficients were computed across all samples using pairwise complete observations. Single-sample gene set enrichment analysis (ssGSEA) was conducted to quantify pathway activity in both dFLEx CDS tumors and human CDS. For dFLEx CDS tumors, mouse

Ensembl gene identifiers were mapped to gene symbols using the org.Mm.eg.db package, and duplicated gene symbols were collapsed by averaging expression values. The resulting gene expression matrix (genes x samples) was used as input for ssGSEA. For human CDS cell lines, gene identifiers were cleaned to remove version suffixes, and the matrix was transposed to (genes x samples). ssGSEA scores were computed using the GSVA package. To assess conservation of pathway activity between species, mean ssGSEA scores for each pathway were compared between mouse tumors and human CDS cell lines using Pearson correlation.

Western blot

Cell lines were maintained in standard growth media until 80% confluency. Using Trypsin-EDTA, the cells were lifted, collected in phosphate-buffered saline (PBS), then pelleted by centrifugation (300xg for 3 minutes). Lysates were made using Pierce RIPA buffer (Thermo Fisher Scientific, Cat. 89900) supplemented with 1% SDS, Halt protease inhibitor (Thermo Fisher Scientific, Cat. 78441), Benzonase, and PhosSTOP phosphatase inhibitor (Roche, Cat. 4906845001), then protein was quantified using Pierce BCA protein assay kit (Thermo Fisher Scientific, Cat. 23225). Heat-denatured proteins were loaded onto a 4-20% Tris-glycine gel, run at 100 V in 1x Tris/Glycine/SDS buffer, and then wet-transferred at 350 mA for 1 hour at 4 °C. When detecting the CIC::DUX4 fusion, all above steps were completed in a single day due to the unstable nature of the fusion protein. CIC::DUX4 (~260 kDa) was probed using anti-DUX4 antibody (Abcam, ab124699, 1:1000), anti-HA antibody (Cell signaling, 3724, 1:1000) or anti-CIC antibody (Origene, AP50924PU-N, 1:1000). RPB1 and p-RPB1 ser2 and ser5 were detected using anti-RPB1 (Cell signaling, 54020, 1:1000) and anti-p-RPB1 antibody (Cell signaling, 54020, 1:1000). gH2AX was detected using gH2AX antibody (Cell signaling, 2577, 1:1000)

with GAPDH (Cell signaling, 2118, 1:4000) or B-actin (Cell signaling, 3700, 1:4000) as loading controls. Images were acquired on a LI-COR Odyssey CLx and processed using Image Studio Software.

Single-nucleus RNA sequencing

Sample processing, library preparations, and sequencing reactions were conducted at GENEWIZ (South Plainfield, NJ, USA). Nuclei extraction was performed using the Miltenyi Nuclei Extraction Buffer (Miltenyi Biotec, Auburn, CA, USA) following the manufacturer's guidelines with gentle MACS Dissociation and C Tubes. Upon isolation, the nuclei were counted using AO/PI dye on the Nexcelom Cellaca MX. Single nuclei RNA libraries were generated using the Chromium Single Cell 3' kit using on-chip multiplexing (10X Genomics, CA, USA). Loading was performed to target capture of ~5,000 GEMs per sample for downstream analysis, and samples were processed through the Chromium Controller following the standard manufacturer's specifications. The sequencing libraries were evaluated for quality on the Agilent TapeStation (Agilent Technologies, Palo Alto, CA, USA), and quantified using the Qubit 2.0 Fluorometer (Invitrogen, Carlsbad, CA). Pooled libraries were quantified using qPCR (Applied Biosystems, Carlsbad, CA, USA) prior to loading onto an Illumina sequencing platform. The sequencing libraries were multiplexed and clustered onto a flowcell on the Illumina NovaSeq X Plus instrument according to the manufacturer's instructions. The samples were sequenced at a configuration compatible with the recommended guidelines as outlined by 10X Genomics. Image analysis and base calling were conducted by the NovaSeq Control Software (NCS). Raw sequence data (.bcl files) generated from Illumina NovaSeq were converted into FASTQ files and demultiplexed using Illumina bcl2fastq 2.20 software. One mismatch was allowed for index

sequence identification. FASTQ reads were aligned to the mm39 reference using cellranger 7.0.1 with default parameters, enabling inclusion of intronic reads. The cellranger output filtered_feature_bc_matrix was utilized for downstream processing to exclude non-cell associated GEMs. After empty droplet removal and initial filtering, mitochondrial genes were identified based on gene annotations from AnnotationHub (Ensembl release 95, *Mus musculus*). MiQC was used to model the relationship between the number of detected genes and mitochondrial read proportion, then cells were filtered based on posterior probability thresholds (default cutoff = 0.75), and an additional threshold requiring ≥200 detected genes per cell. Library size normalization was performed using the deconvolution method implemented in scran and log-normalized expression values were calculated using logNormCounts from scuttle. After identifying the top 2000 highly variable genes (HVGs), principal component analysis (PCA) was performed using the scater package. The processed and normalized SingleCellExperiment object was saved as an RDS file for downstream analyses.

Single-nucleus RNA sequencing data analysis

Single-nucleus RNA-seq data were processed and analyzed in R using the SingleCellExperiment, scater, and scran packages. Doublets were detected and filtered using scDblFinder, then Harmony integration was used to batch correct across samples. Unsupervised clustering was performed using the scran::clusterCells with a shared nearest neighbor graph (SNN) (k = 40) and Louvain community detection (resolution = 0.4). To define cluster-specific marker genes, scran::findMarkers was used to compare each cluster to all other clusters. Genes were then ranked by false discovery rate (FDR) and log fold change. Cell type annotation was assigned based on canonical marker gene expression. Marker gene sets representing major cell

populations (macrophages, fibroblasts, endothelial cells, myocytes, neural crest cells, T cells, B cells, neutrophils, dendritic cells, mesenchymal cells, NK cells, pericytes, muscle stem cells, neural cells, and CIC::DUX4 tumor cells) were curated and used to guide annotation. Cluster identities were manually assigned based on enrichment of these markers and visual inspection of UMAP expression patterns. To further characterize CIC::DUX4 tumor cells, subclustering was conducted on tumor cells using SNN graph (k = 20) with Louvain clustering (resolution = 0.4). To define marker genes for the tumor subclusters, scran::findMarkers was again used. To infer the developmental potential of tumor cells, CytoTRACE2 was conducted as previously described (29). Briefly, raw count matrices were extracted and subsampled to a maximum of 6,000 cells for computational efficiency. CytoTRACE2 scores were computed using default parameters for mouse data, and CytoTRACE2 scores per tumor subcluster were averaged to assess relative stemness. To assess CIC::DUX4 activity, a transcriptional activity score was calculated by averaging log-normalized expression values of known target genes (*Etv4, Etv1, Etv5, Shc3, Shc4, Pole, Dusp4, Vgf, Ccnd2, Ccne1, Muc5ac, Spred2, Spred1, Myc,* and *Ets1*). Scores were computed per cell and compared across tumor subclusters. To investigate whether tumor subclusters resemble specific cell lineages, tumor cells were compared to the Mouse Organogenesis Cell Atlas (MOCA) (24). MOCA count matrices were aggregated by annotated developmental trajectories and normalized to log-transformed counts per million (CPM). Tumor cells were pseudobulked by subcluster, normalized, and matched to MOCA data using shared genes. Spearman and Pearson correlation analyses were performed between tumor subclusters and MOCA trajectories. To improve specificity, ribosomal and mitochondrial genes were excluded, and expression values were z-scored per gene prior to correlation. Relative correlation

scores were calculated by subtracting row means, and the highest-correlating trajectory was assigned to each tumor subcluster.

Cell Culture

DMEM (Cat. 11995065), fetal bovine serum (FBS, Cat. A3160702) and penicillin/streptomycin (P/S, Cat. 15140122) were purchased from Gibco, RPMI was from Sigma-Aldrich (Cat. R8758). Kitra-SRS (CIC::DUX4 sarcoma cell line gifted by Satoshi Takenaka from Osaka University Graduate School of Medicine, Osaka, Japan (17)), TOPCDS (mouse CIC::DUX4 sarcoma cell line (22)), A-673 (Ewing sarcoma cell line, ATCC), and WT MEFs were cultured in DMEM/10% FBS/P/S. ECD1 (CIC::DUX4 sarcoma cell line gifted by Takuro Nakamura from the Japanese Foundation for Cancer Research, Tokyo, Japan (7)), X1C1 (CIC::DUX4 sarcoma cell line gifted by Tadashi Kondo from the National Cancer Center Research Institute, Tokyo, Japan (18)), CDS2 (CIC::DUX4 sarcoma cell line gifted by Tadashi Kondo from the National Cancer Center Research Institute, Tokyo, Japan (20)), and Rh-4 (fusion-positive Rhabdomyosarcoma cell line gifted by Corinne Linardic from Duke University, Durham, NC) were cultured in RPMI/20% FBS/P/S. All cells were cultured at 37 °C in a 5% CO_2 atmosphere. Importantly, FBS was not heat-inactivated to preserve phosphatase activity required for the conversion of Minnelide to triptolide *in vitro.*

Cell Viability (CellTiter-Glo) assay

Cell lines were plated at a density of 3-5 x 10^4 cells per well in 96-well plates. In technical quadruplicates, cells were incubated in Minnelide (MedChemExpress, HY-124584,

dissolved in H_2O) at concentrations ranging from 0nM to 200nM for 48h. Cell viability was assayed using CellTiter-Glo luminescence (Promega, G7572). The plates were read using a CLARIOstar Plus plate reader, and RLU was normalized to 0nM untreated cells.

EdU Incorporation assay

Cells were grown to 70% confluence on 12 mm poly-L-lysine coated glass coverslips (Neuvitro, GG-12-15-PLL) and treated with Minnelide (MedChemExpress, HY-124584) at 25nM concentration or vehicle for 48 h. 5-ethynyl-2'-deoxyuridine (EdU) incorporation assay was carried out according to manufacturer's instructions (Click-iT™ Plus EdU Cell Proliferation Kit for Imaging, Alexa Fluor™ 647 dye, Invitrogen, C10640). Briefly, cells were incubated with 10 µM EdU for 1 h prior to fixation in 3.7% PFA for 15 min at RT Following this, the cells were washed in 3% BSA and permeabilized using 0.5% Triton X-100 for 20 min at RT. Cells were then incubated in Click-iT detection reagents (Alexa Fluor 647, 1:1000) for 30 min in the dark at RT, followed by nuclear staining using 1 ug/mL DAPI. Coverslips were mounted on SuperFrost slides (Fisher Scientific, 22037246) using ProLong Gold Antifade mounting media (Invitrogen, P36930). All images were taken using a Leica Stellaris 5 confocal microscope equipped with an HC PL APO 20x/0.75 NA CS2 objective. Frequency of EdU-positive cells was calculated on Fiji using manual thresholding parameters.

Epoxomicin and Minnelide co-treatment

Cell lines were plated at a density of 1 x 10^6 cells per 10cm dish. The following day, 10uM epoxomicin (MedChemExpress, HY-13821) and/or 10uM Minnelide (MedChemExpress, HY-

124584) was added to the cells for 120 minutes. Because inhibition of the proteasome for either the full 18-24h of Minnelide treatment or for 6-8h during Minnelide treatment is very toxic for the cells, these experiments were conducted using a high concentration of Minnelide over shorter time periods (120 minutes), as was previously shown in SK-OV-3 ovarian cells (50).

Generation of XPB mutant

The XPB C342T mutant construct was generated by GeneScript. For lentiviral production, 293FT cells were transfected with XPB_Cys342Thr_pGenLenti, pRSV-Rev, pMDLg/pRRE, and pMD2.G using Lipofectamine 2000 (Thermo Fisher Scientific). Kitra-SRS and ECD1 cells were transduced with viral supernatant using a concentration of 2 ug/mL polybrene, followed by antibiotic selection using 0.25-0.5mg/mL puromycin.
