# Supplementary material for: Targeting CIC::DUX4 sarcoma with Minnelide in a dual recombinase–initiated genetically engineered mouse model
Source: J Clin Invest. 2026 Jun 16;136(14):e202218. doi: 10.1172/JCI202218 (PMC13367964; doi:10.1172/JCI202218)

Full Unedited blot for Figure 4B

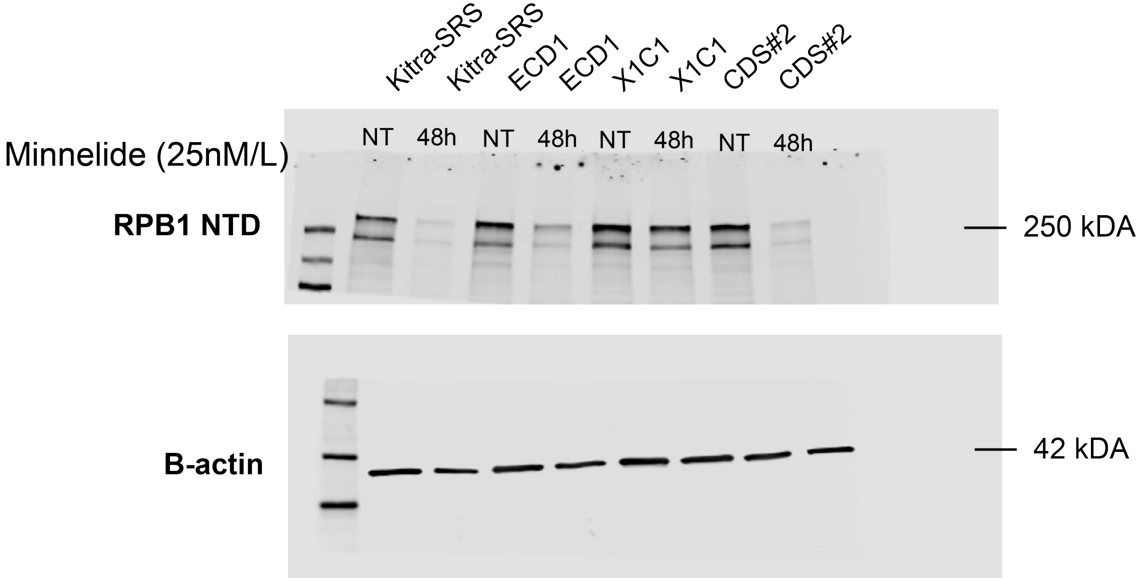

Full Unedited blots for Figure 4C **ECD1**

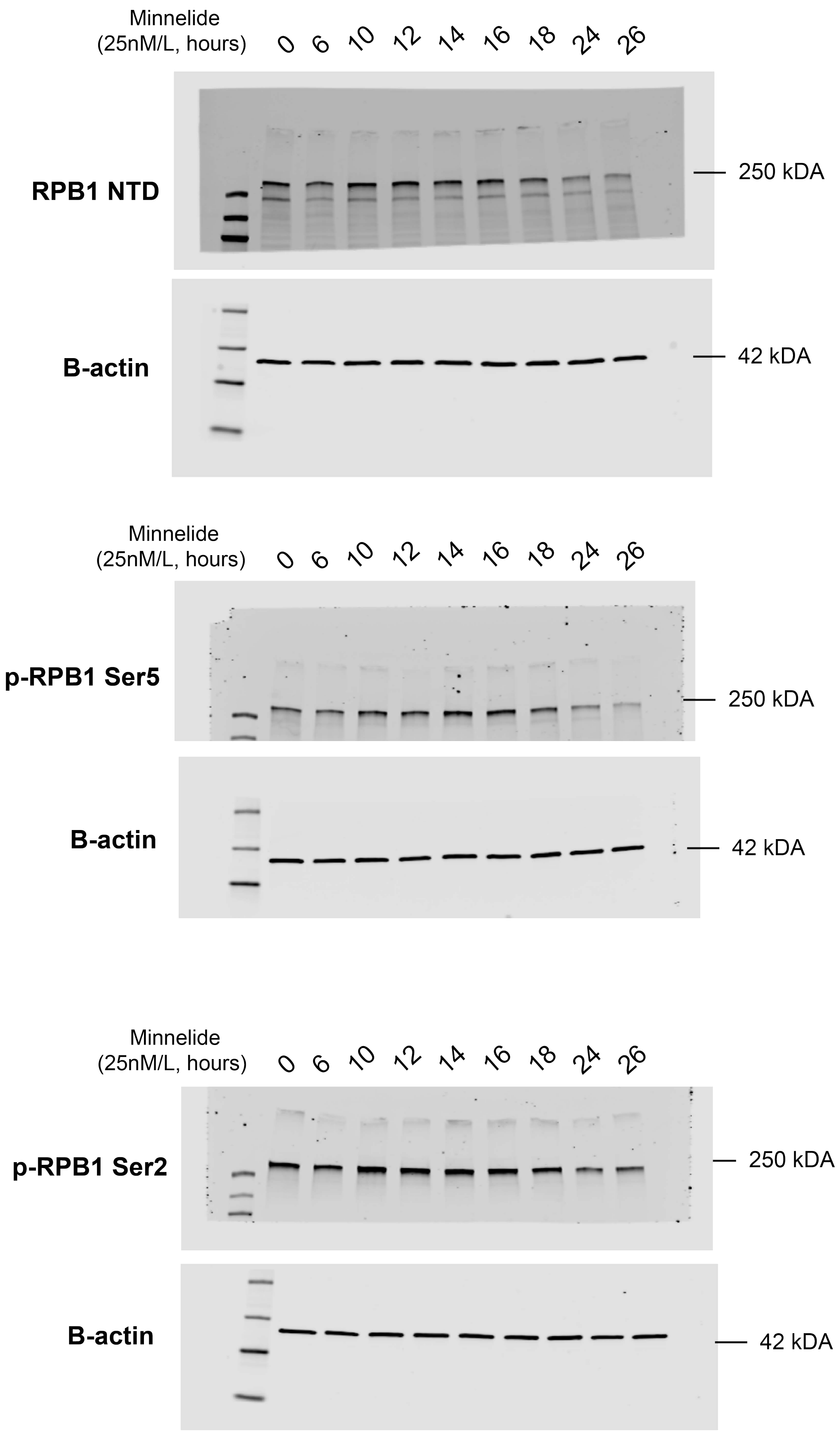

Full Unedited blots for Figure 4E

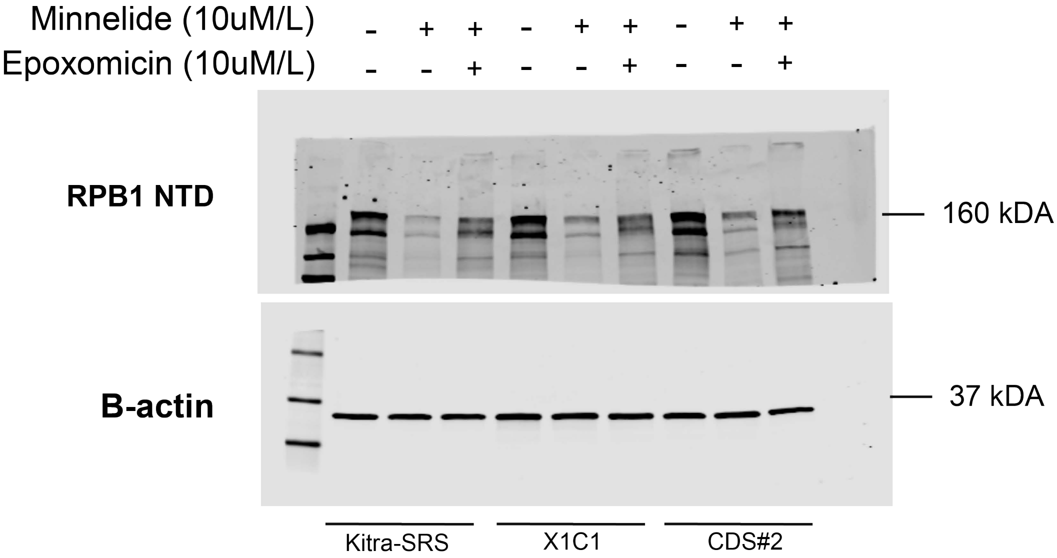

Full Unedited blots for Figure 4F

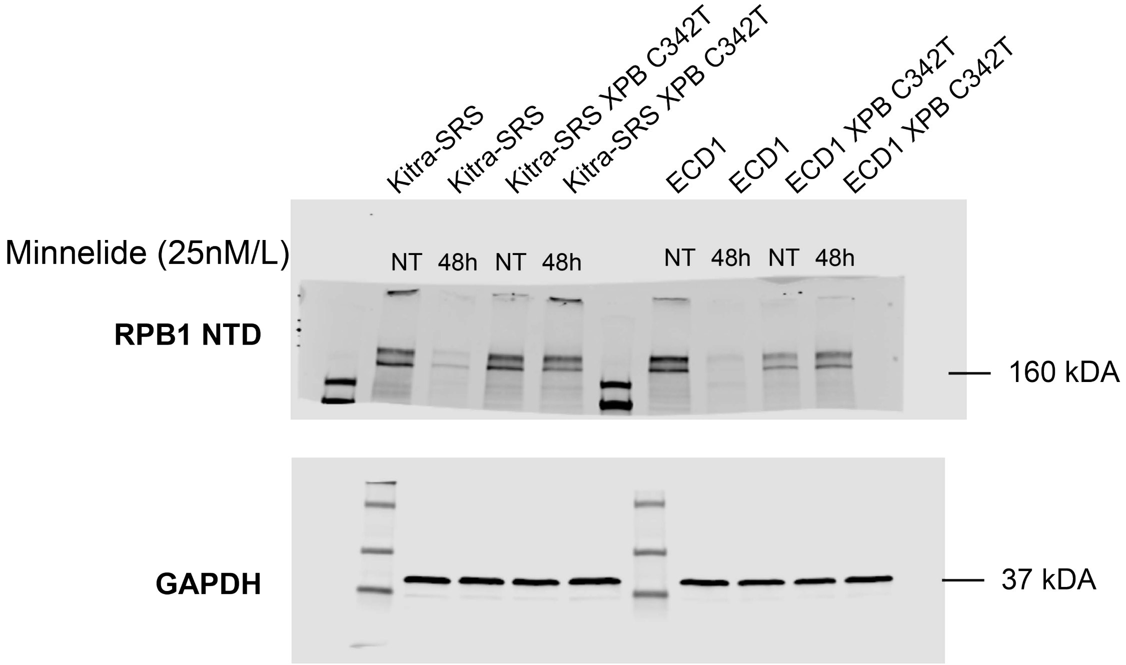

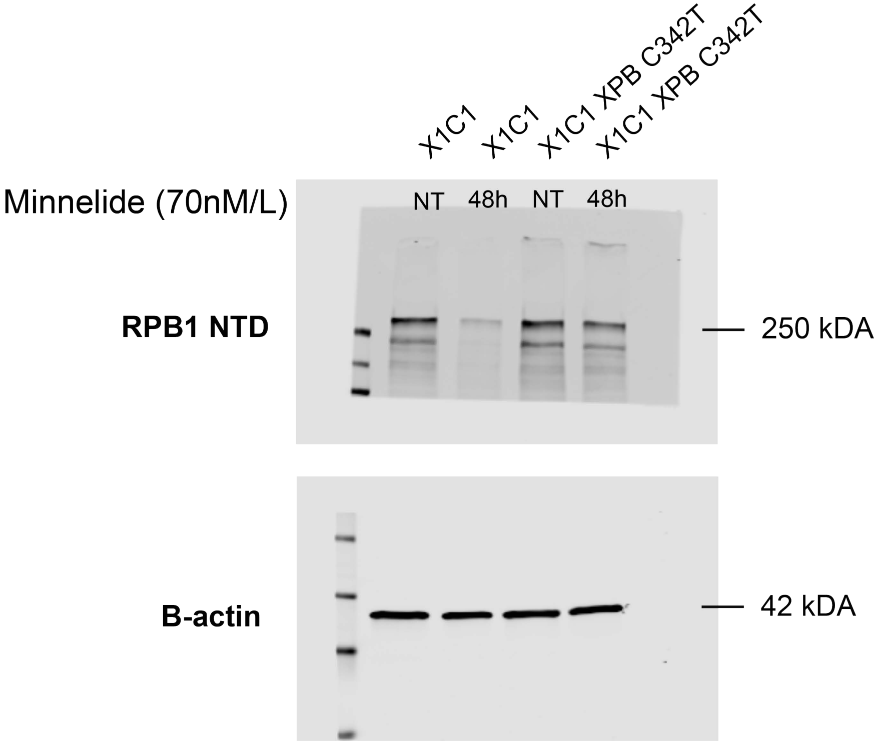

Full Unedited blots for Figure 6E

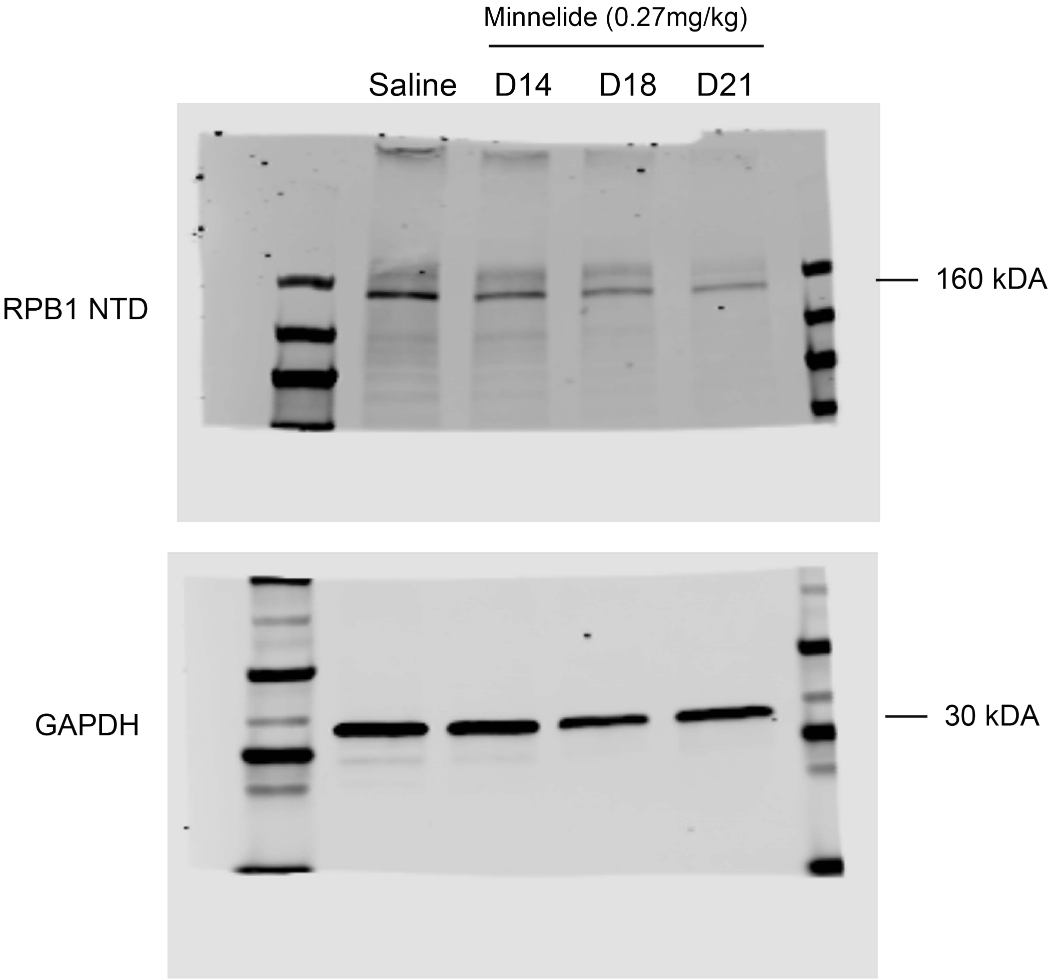

Full Unedited blots for Supplementary Figure 1M

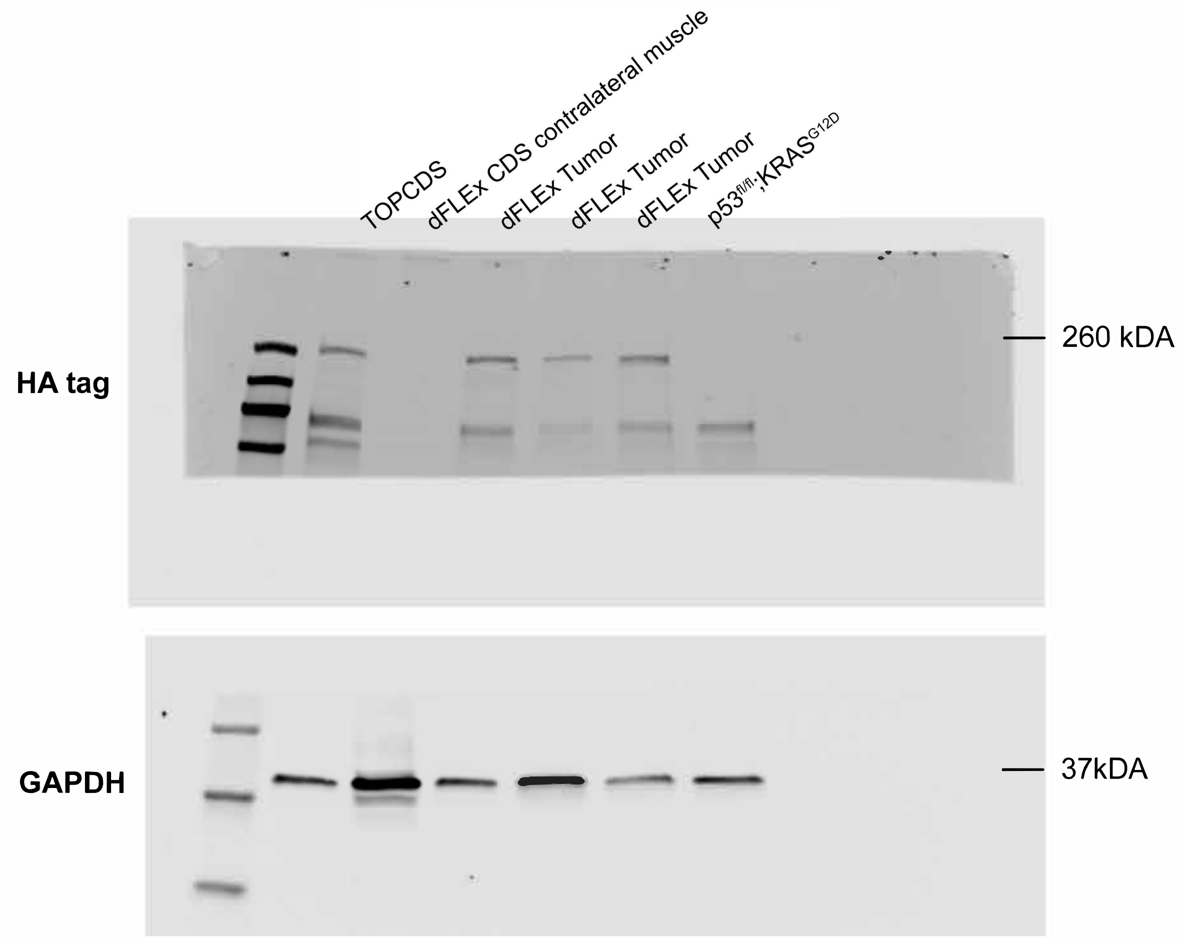

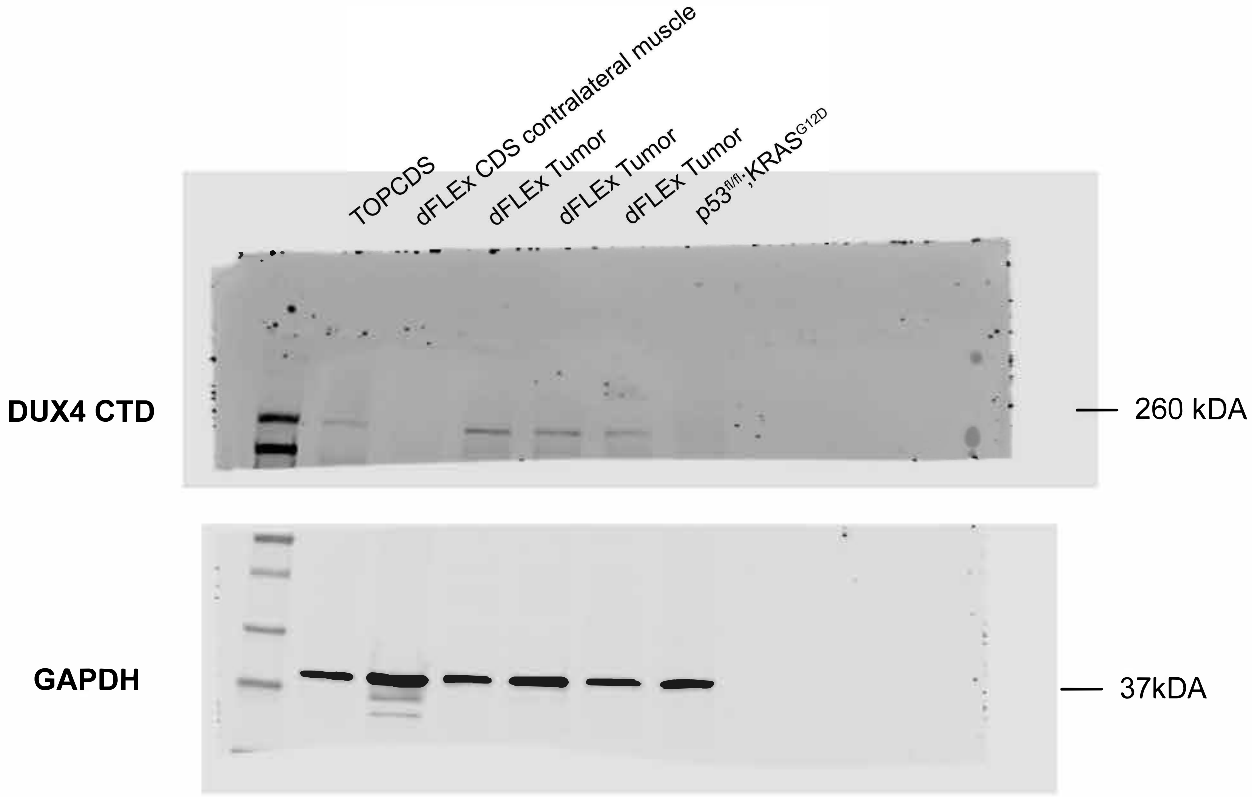

Full Unedited blots for Supplementary Figure 4A

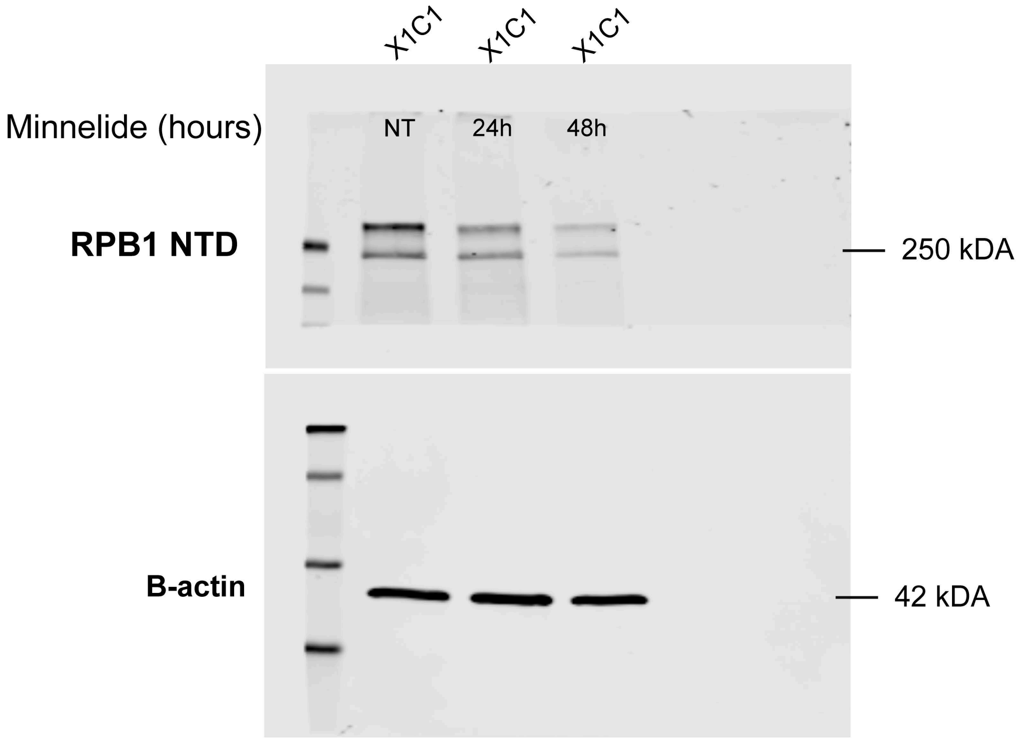

Full Unedited blots for Supplementary Figure 4B

Kitra-SRS

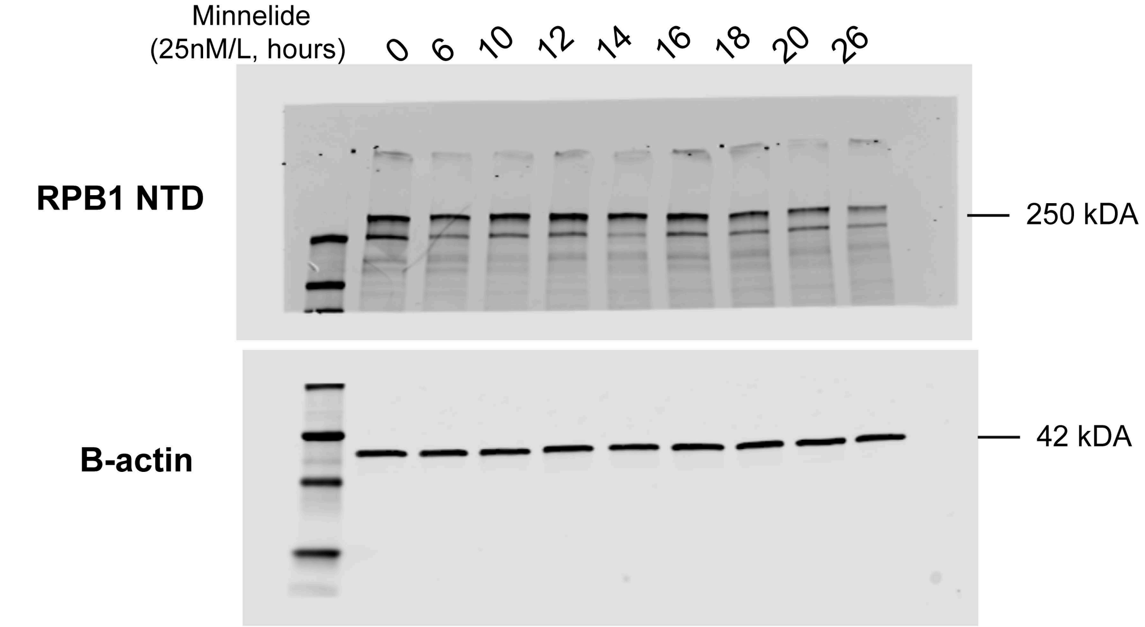

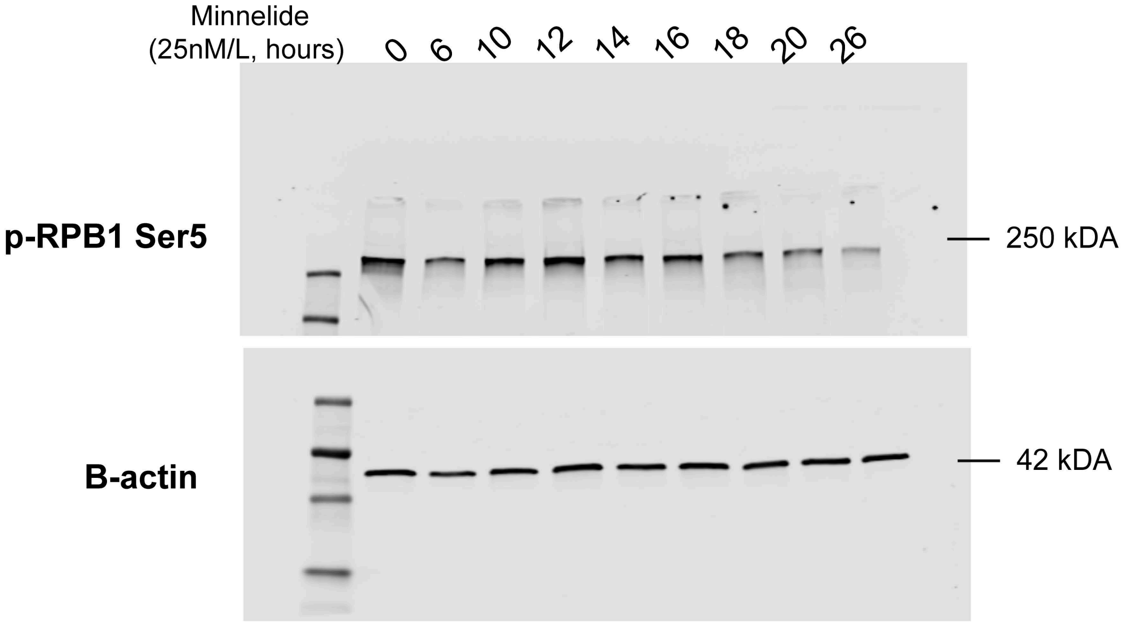

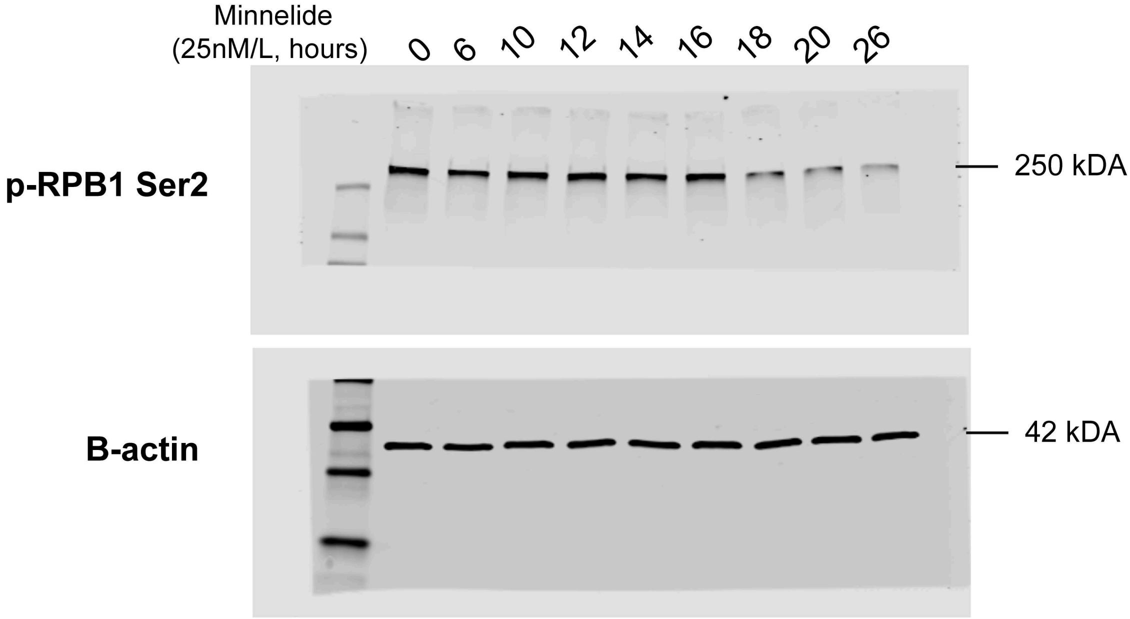

Full Unedited blots for Supplementary Figure 4B

CDS#2

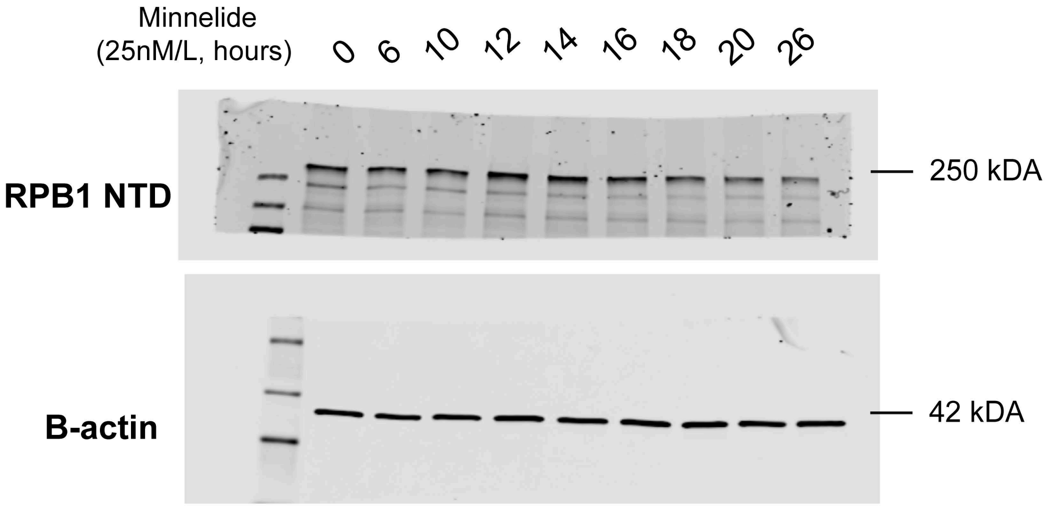

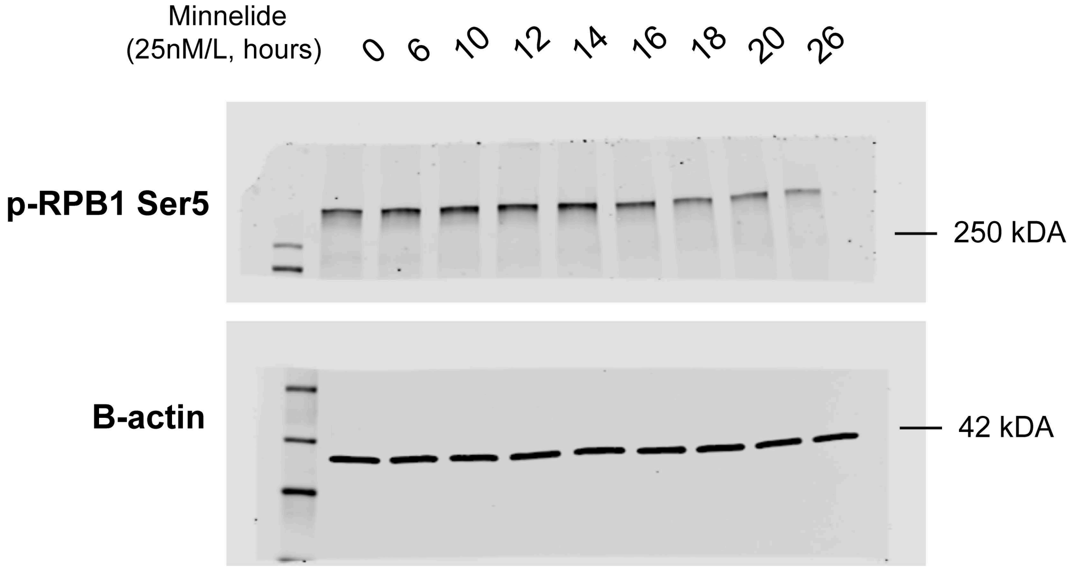

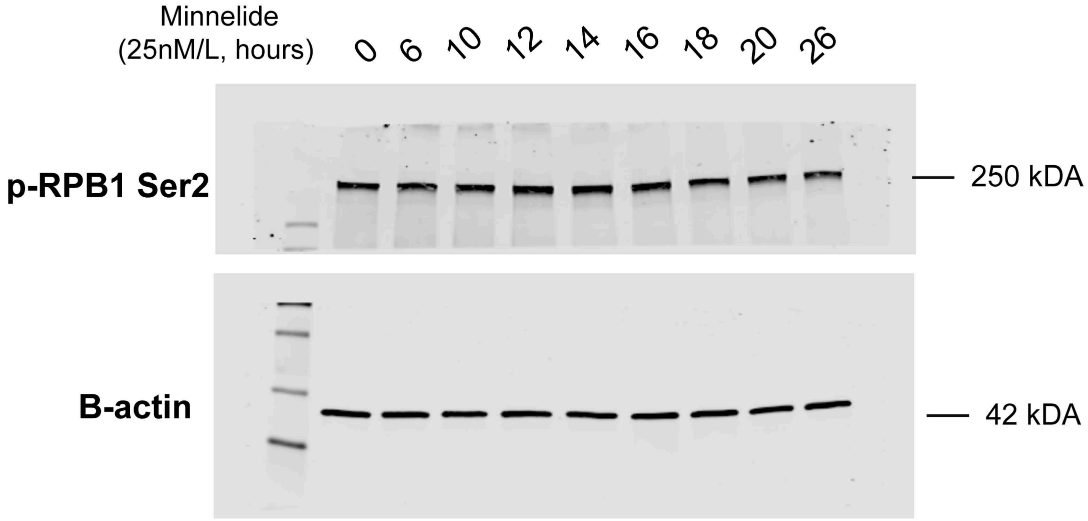

Full Unedited blots for Supplementary Figure 4B **X1C1**

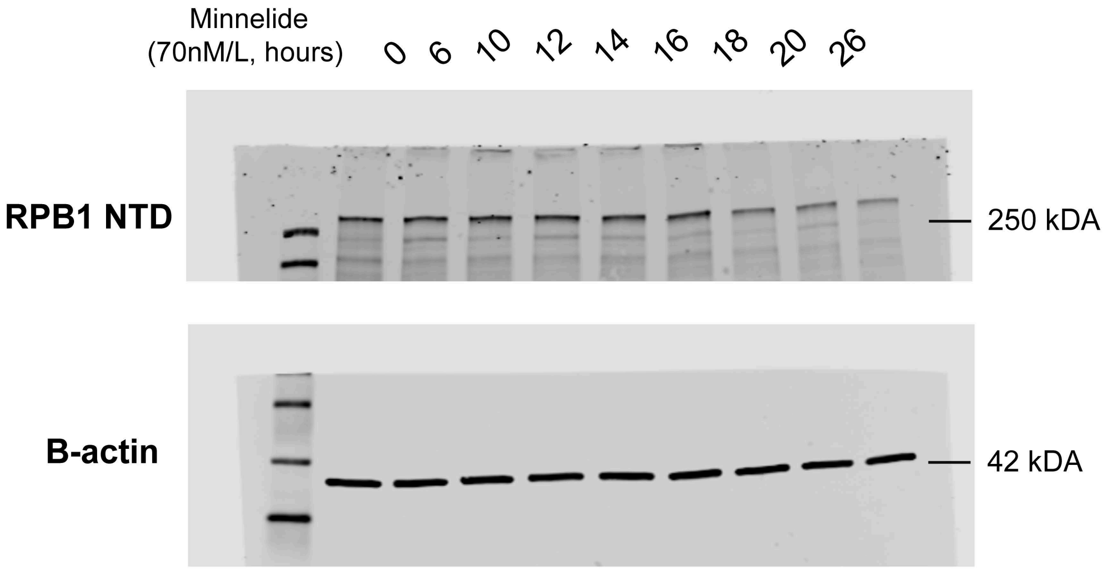

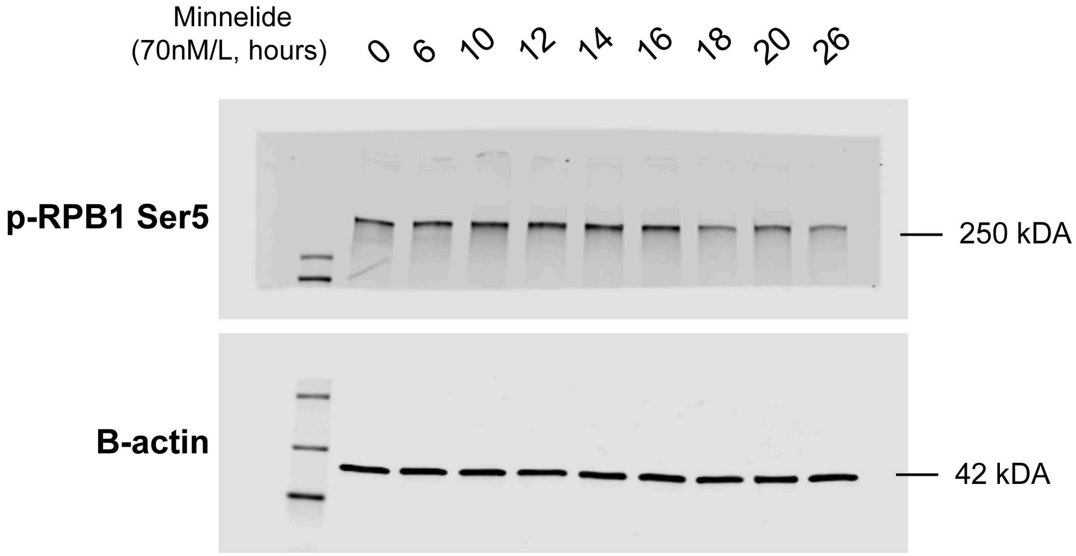

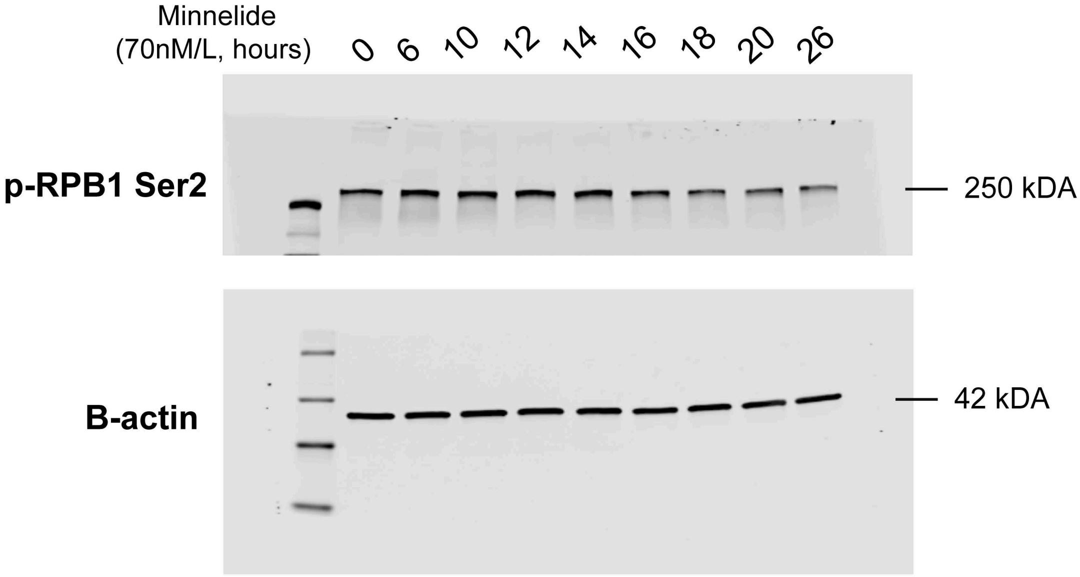

Full Unedited blots for Supplementary Figure 4C

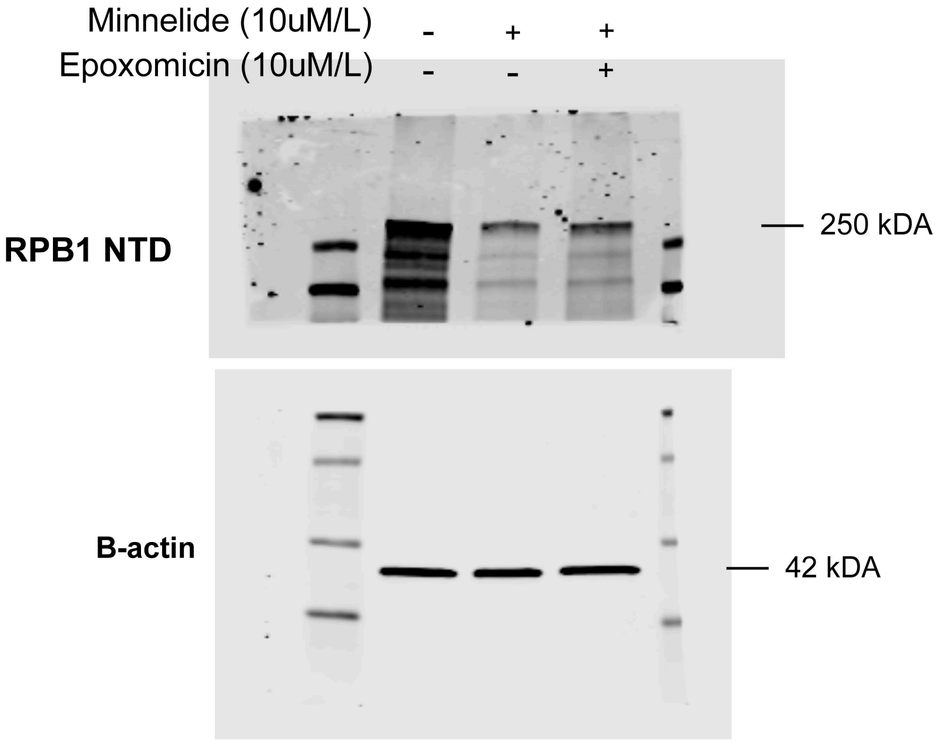

Full Unedited blots for Supplementary Figure 4F

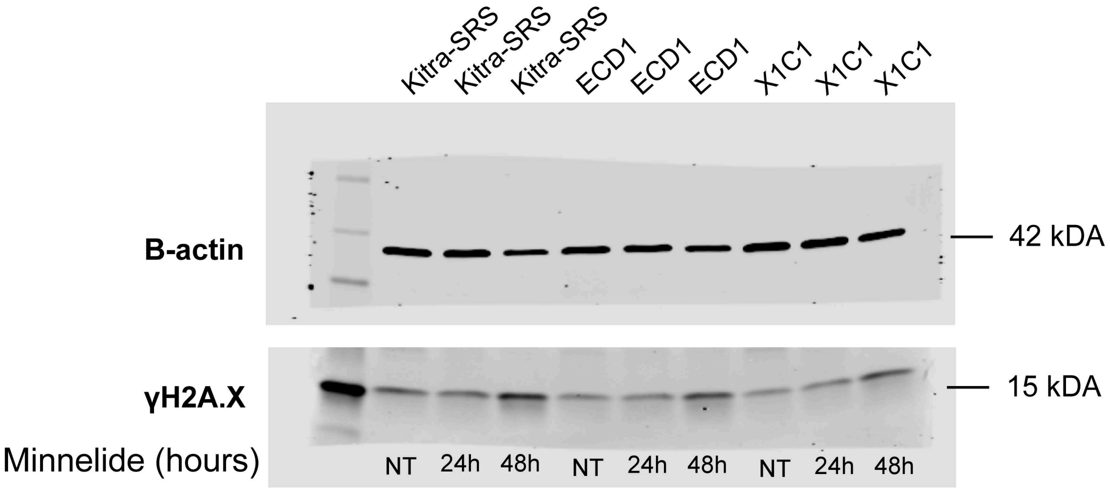

Full Unedited blots for Supplementary Figure 5B

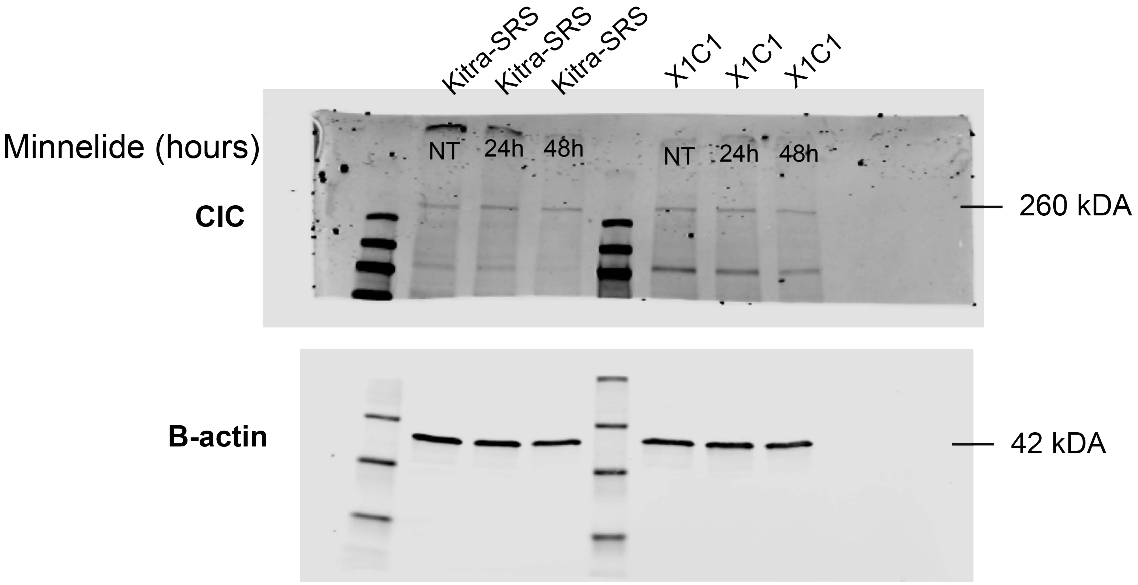

Supplement: Unedited blot and gel images [file jci-136-202218-s276.pdf]
